# Actin activates *Pseudomonas aeruginosa* ExoY nucleotidyl cyclase toxin and ExoY-like effector domains from MARTX toxins

**DOI:** 10.1038/ncomms13582

**Published:** 2016-12-05

**Authors:** Alexander Belyy, Dorothée Raoux-Barbot, Cosmin Saveanu, Abdelkader Namane, Vasily Ogryzko, Lina Worpenberg, Violaine David, Veronique Henriot, Souad Fellous, Christien Merrifield, Elodie Assayag, Daniel Ladant, Louis Renault, Undine Mechold

**Affiliations:** 1 Institut Pasteur, CNRS UMR3528, Unité de Biochimie des Interactions Macromoléculaires, Département de Biologie Structurale et Chimie, 25-28 rue du Docteur Roux, 75724 Paris cedex 15, France; 2 Department of Bacterial Infections, Gamaleya Research Center, Moscow 123098, Russia; 3 Institut Pasteur, CNRS UMR3525, Génétique des Interactions Macromoléculaires, Département de Génomes et Génétique, 25-28 rue du Docteur Roux, 75724 Paris cedex 15, France; 4 Institut Gustave Roussy, CNRS UMR 8126, Unité de Signaling, Nuclei and Innovations in Oncology, 94805 Villejuif, France; 5 Institute for Integrative Biology of the Cell (I2BC), CEA, CNRS, Univ. Paris-Sud, Université Paris-Saclay, 91198 Gif-sur-Yvette cedex, France

## Abstract

The nucleotidyl cyclase toxin ExoY is one of the virulence factors injected by the *Pseudomonas aeruginosa* type III secretion system into host cells. Inside cells, it is activated by an unknown eukaryotic cofactor to synthesize various cyclic nucleotide monophosphates. ExoY-like adenylate cyclases are also found in Multifunctional-Autoprocessing Repeats-in-ToXin (MARTX) toxins produced by various Gram-negative pathogens. Here we demonstrate that filamentous actin (F-actin) is the hitherto unknown cofactor of ExoY. Association with F-actin stimulates ExoY activity more than 10,000 fold *in vitro* and results in stabilization of actin filaments. ExoY is recruited to actin filaments in transfected cells and alters F-actin turnover. Actin also activates an ExoY-like adenylate cyclase MARTX effector domain from *Vibrio nigripulchritudo*. Finally, using a yeast genetic screen, we identify actin mutants that no longer activate ExoY. Our results thus reveal a new sub-group within the class II adenylyl cyclase family, namely actin-activated nucleotidyl cyclase (AA-NC) toxins.

## Abstract (web summary)

The ExoY toxin is injected by *Pseudomonas aeruginosa* into host cells, where it is activated by an unknown host factor. Here the authors identify such factor as filamentous actin.

## Graphical abstract

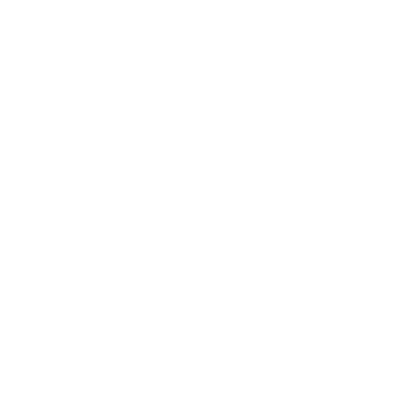

P*seudomonas aeruginosa* is an opportunistic human pathogen that causes severe infections in immune-compromised individuals and is a major cause of chronic infections in cystic fibrosis patients. Equipped with a type III secretion system (T3SS), *P. aeruginosa* can inject effector proteins directly into host cells where they contribute to virulence of the pathogen (for reviews see refs 1, 2). Four different T3SS-delivered effectors have been characterized (exoenzyme T, Y, U and S), but new effectors were recently identified^3^. Exoenzyme Y (ExoY) is present in 89% of clinical isolates^4^. It was originally identified as an adenylate cyclase in 1998 due to amino-acid sequence homology with two well-characterized class II adenylate cyclase toxins, CyaA from *Bordetella pertussis* and edema factor from *Bacillus anthracis*^5^. Recent studies employing cultured cells revealed that substrate specificity of these enzymes is not restricted to ATP: edema factor and CyaA were shown to use uridine-5′-triphosphate (UTP) and cytidine-5′-triphosphate (CTP) as substrate^6^ while ExoY was shown to promote the intracellular accumulation of several cyclic nucleotides^7^,^8^ with a preference for cyclic GMP (cGMP) and cyclic UMP (cUMP) over cyclic AMP (cAMP) and cyclic CMP (cCMP) formation^7^.

ExoY was shown to induce cell death in a cellular infection model^9^ and severe, long-term lung damage in an animal infection model in rats^10^. At the molecular level, ExoY was associated with microtubule breakdown causing the formation of gaps between endothelial cells and increased permeability of the endothelial barrier^8^,^11^,^12^,^13^. These effects were, however, not seen in all studies^14^,^15^ and could be attributed to different expression levels of ExoY, as well as the use of different bacterial strain backgrounds and cell lines.

Recent whole-genome sequencing projects have identified ExoY nucleotidyl cyclase modules among a variety of toxic Multifunctional-Autoprocessing Repeats-in-ToXin (MARTX) effector domains in numerous bacterial species of the *Vibrio* genus^16^ that represent emerging human or animal pathogens. These ExoY-like domains can be essential for virulence^16^. Elucidating the enzymatic specificities and molecular mechanisms of pathogenicity of ExoY and ExoY-like toxins may, therefore, help finding new therapeutic strategies against the toxicity and virulence of several bacterial pathogens.

Despite the progress in understanding downstream effects of ExoY activity, fundamental information on ExoY is lacking: similar to other bacterial soluble related cyclases such as CyaA and edema factor, ExoY is inactive in bacteria and is activated by an unknown eukaryotic cofactor after its delivery to the target cells^5^. Whereas the other class II adenylate cyclase toxins such as CyaA and edema factor are strongly activated upon interaction with calmodulin^17^,^18^, calmodulin is unable to stimulate ExoY enzymatic activity and the precise nature of the eukaryotic activator has remained elusive up to now. Here we report the identification of actin as the cofactor that activates *P. aeruginosa* ExoY and the ExoY-like module present in MARTX toxin of *Vibrio nigripulchritudo* in host cells. Our findings suggest that actin is the common eukaryotic activator for a sub-group of the class II adenylyl cyclase toxin family^19^.

## Results

### An activator of ExoY is present in *Saccharomyces cerevisiae*

**Figure 1:**
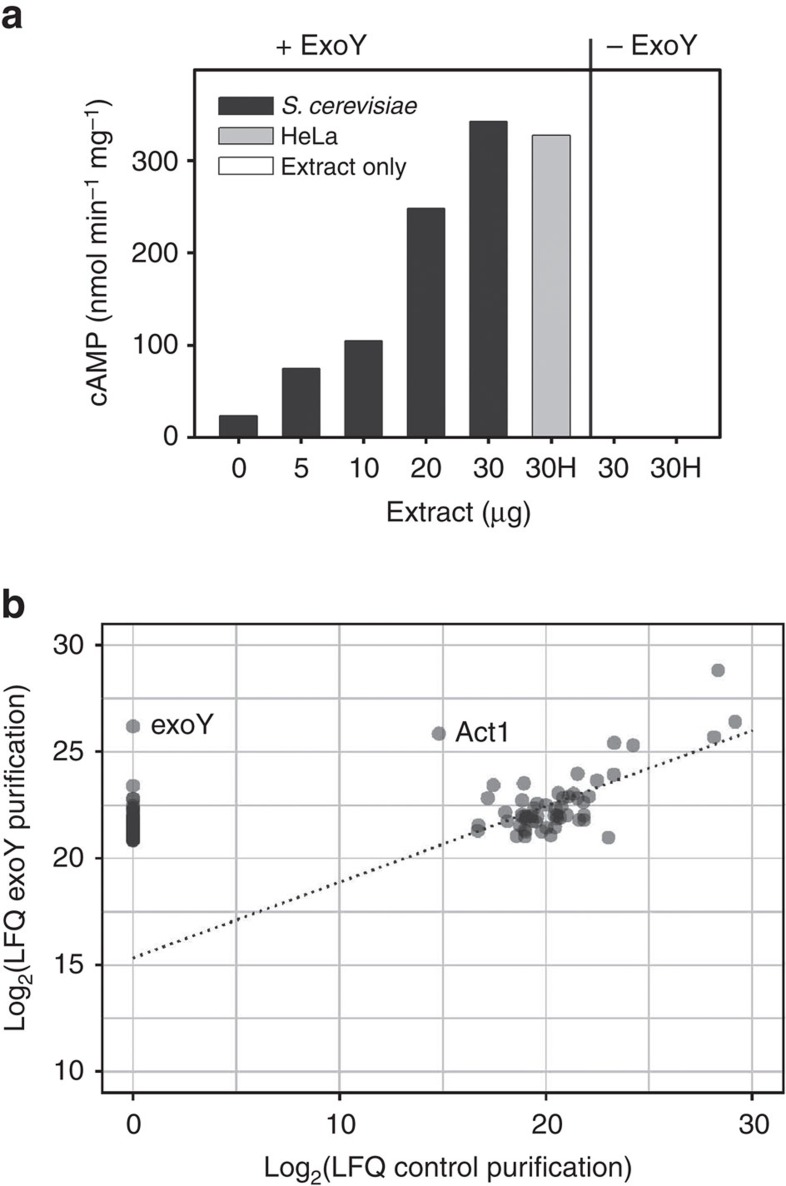
Presence of an activator of ExoY in *Saccharomyces cerevisiae*. (**a**) Activation of HF-ExoY by extracts from HeLa cells or *S. cerevisiae*. Reactions (50 μl) containing 1 μg ExoY were started by the addition of 2 mM ATP substrate and stopped after 30 min incubation at 30 °C and the amount of synthesized cAMP was measured. (**b**) Specific association of yeast Act1 to ExoY^K81M^. Log_2_ transformed LFQ scores for the proteins identified in the fraction that copurified with ExoY^K81M^-TAP (*y* axis) were represented as a function of the scores obtained for the control purification (ExoY^K81M^-HA, *x* axis). Black circles are the result of two or more superimposed grey circles. For clarity, only the 100 proteins with highest LFQ scores in the TAP purification are shown. Forty-five of these factors, including ExoY, were not identified in the control purification and are represented on the *y* axis alone. The dashed line was computed by linear regression for the 55 proteins having LFQ values in both experiments and indicates the trend for common contaminants in the affinity purification.

Arnoldo *et al*.^20^ have reported that overexpression of ExoY impairs yeast growth, suggesting that ExoY is active in this organism and, therefore, that a cofactor required for ExoY catalytic activity should be present in yeast. To test this hypothesis, we prepared extracts from *Saccharomyces cerevisiae* BY4741 cells and measured adenylate cyclase activity of recombinant ExoY carrying an N-terminal His-Flag tag (HF-ExoY) in the presence of increasing amounts of yeast cell extracts *in vitro*. Extracts from HeLa cells were used as positive control. We observed a dose-dependent stimulation of ExoY activity by yeast cell extracts, to levels that were similar to those measured when using HeLa cell extracts (Fig. 1a). Thus, we decided to use *S. cerevisiae* as a convenient experimental system to identify the putative yeast activator that was likely to be evolutionarily conserved in human cells.

### Actin interacts with ExoY-TAP in *S. cerevisiae*

To identify putative ExoY-binding proteins in yeast, plasmid-encoded ExoY with either a C-terminal TAP- or HA-tag (ExoY-TAP or ExoY-HA, respectively) was expressed in *S. cerevisiae.* To avoid toxic effects due to cyclic nucleotide accumulation, we used a catalytically inactive variant of ExoY, ExoY^K81M^ (in which the Lys81 was mutated to Met^5^). Proteins co-purifying with the affinity purified bait protein were isolated by affinity purification on rabbit IgGs covalently bound to magnetic beads and analysed by SDS–polyacrylamide gel electrophoresis (PAGE) (Supplementary Fig. 1), or processed by tryptic digestion and liquid chromatography–mass spectrometry (LC–MS/MS) peptide/protein analysis. The raw data were then analysed by MaxQuant for protein identification and quantitative estimation of the specific enrichment of proteins in the experimental sample (ExoY^K81M^-TAP) as compared with the control (ExoY^K81M^-HA). While many abundant proteins were present in both samples to a similar degree, as estimated from the label-free quantitation score (LFQ^21^), ExoY was identified exclusively in the purification performed with ExoY^K81M^-TAP extracts, as expected. Another protein that was about 1,000 times more abundant in the ExoY^K81M^-TAP purification than in the control was yeast actin (Uniprot P60010, YFL039C, Act1), which showed an LFQ score close to the score of the tagged ExoY (Fig. 1b). Other factors were identified specifically in the ExoY^K81M^-TAP purification, but with much lower LFQ scores (see Supplementary Data 1). These results suggested a specific interaction of ExoY^K81M^ with actin. Since actin is both specific to eukaryotic cells and one of the most highly conserved and abundant proteins in these cells, actin appeared to be an appropriate candidate for activating ExoY in mammalian cells.

### ExoY interacts with mammalian actin filaments *in vitro*

**Figure 2:**
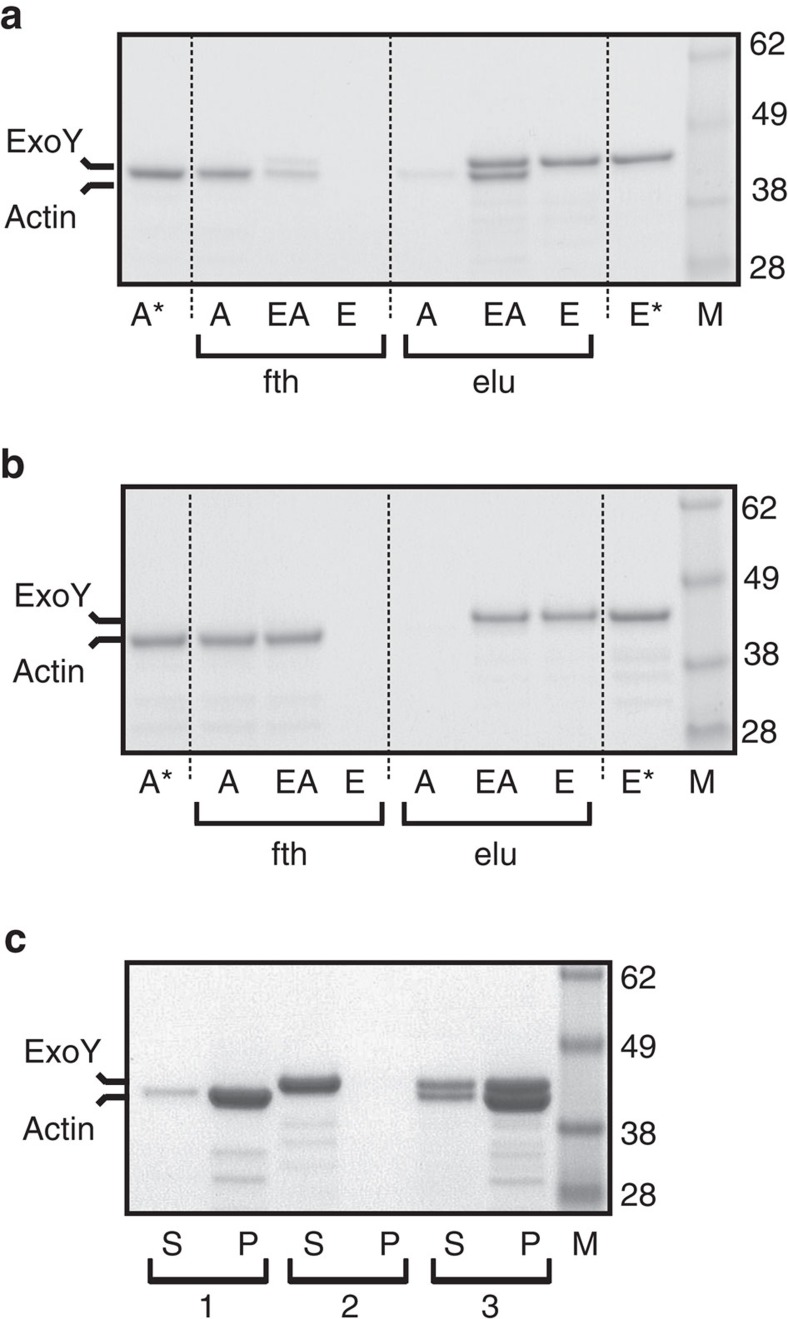
Interaction between ExoY and skeletal muscle actin from rabbit. (**a**,**b**) Actin (MA-L) was converted to Mg-ATP actin and allowed to polymerize to steady state at 20 μM for 2 h at room temperature (**a**) or prevented from polymerization by the addition of latrunculin A to 30 μM (**b**), then diluted fivefold in binding buffer (20 mM imidazole) and added to 7.5 μl Ni-NTA agarose beads. Equimolar amounts (12 μg) of ExoY-FH or the corresponding buffer were added and binding was allowed in batch for 1 h at 4 °C. Samples were washed three times with buffer containing 20 mM imidazole and once in buffer containing 40 mM imidazole before elution in SDS-sample buffer. Aliquots were separated on 4–12% NuPAGE Bis-Tris gels (Invitrogen) in NuPAGE MES SDS running buffer and the gel was stained with Bio-Safe Coomassie stain. Lanes (A): actin only; (E): ExoY only; (AE): both actin and ExoY-FH; (A*) and (E*): corresponding input for actin and ExoY alone, respectively; (fth): flow through, that is, fraction not bound to Ni-NTA beads; (elu): fractions eluted from Ni-NTA agarose. Corresponding amounts were loaded to allow direct comparability. Aliquots correspond to 10% of the total sample volume. The band of lower molecular weight was confirmed to be actin by western blots with anti-actin C4 antibodies. (**c**) Cosedimentation of ExoY with skeletal muscle F-actin (MA-99). Supernatant (S) and pellet (P) fractions were separated on 4–12% NuPAGE Bis-Tris gels (Invitrogen) in NuPAGE MES SDS running buffer and the gel was stained as above. Reaction (1): actin only, (2): ExoY-FH only, (3) actin plus ExoY-FH.

To verify the interaction between ExoY and mammalian actin *in vitro*, we performed Ni-NTA agarose pulldowns using ExoY with a C-terminal Flag-His tag (ExoY-FH) and α-actin from rabbit skeletal muscle. Binding of ExoY to polymerized actin (F-actin, Fig. 2a) or to monomeric actin (G-actin) that was prevented from polymerization by the drug latrunculin A (Fig. 2b) was tested. While no binding of G-actin to ExoY could be observed (EA, Fig. 2b), quantitative densitometry analysis showed that 92% of F-actin and 100% of ExoY could be recovered by elution (elu) from samples that contained both proteins (EA, Fig. 2a). The unspecific binding of F-actin to Ni-agarose beads was low under the conditions tested as 85% of F-actin was found in the unbound fraction (fth) in samples containing only F-actin (A, Fig. 2a).

To confirm ExoY binding to F-actin, we performed high-speed cosedimentation assays using mixtures of ExoY and F-actin polymerized to steady state. High-speed centrifugation separated F-actin present in the pellet from G-actin present in the supernatant. Figure 2c shows that ExoY, which alone partitioned into the supernatant, was mostly found in the pellet fraction in the presence of F-actin, providing additional evidence that ExoY can interact with F-actin.

### Actin stimulates ExoY nucleotidyl cyclase activity *in vitro*

**Figure 3:**
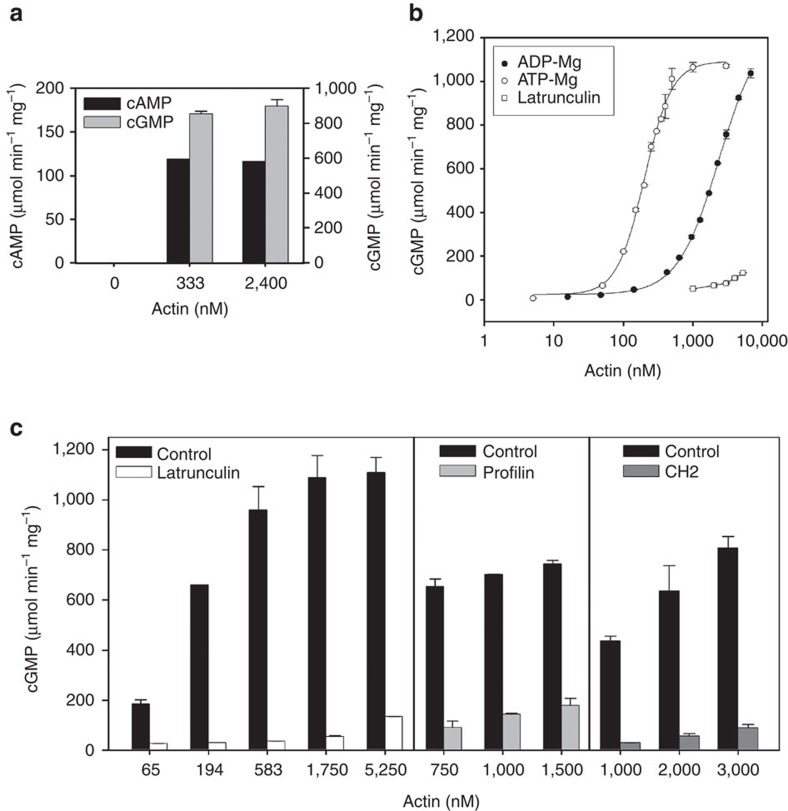
ExoY is efficiently activated by actin *in vitro*. (**a**) Preferential synthesis of cGMP as compared with cAMP by ExoY activated by actin (A-99). Reactions containing HF-ExoY at 0.5 nM (1 ng) and actin at concentrations indicated were started by the addition of 2 mM ATP or GTP substrate and incubated for 10 min at 30 °C. (**b**) Dependence of ExoY activation on the nucleotide and polymerization state of actin. Muscle α-actin (MA-L) was converted to Mg-ATP-actin or Mg-ADP-actin and used at the concentration indicated to activate ExoY-FH. Mg-ATP-actin or Mg-ADP-actin polymerize above 100 and 2,000 nM, respectively. Activities with Mg-ATP-actin monomers saturated by the polymerization-inhibiting drug latrunculin A (according to **c**) were plotted as comparison. (**c**) Effect of latrunculin A and G-actin-binding proteins on the activation of ExoY. Reactions containing actin (+/− Latrunculin A) and ExoY-FH were preincubated for 10 min at 30 °C and started by the addition of 2 mM GTP and continued for 10 min. Latrunculin A: Latrunculin A was present at a twofold excess over actin (MA-L) and was added to actin and preincubated for 10 min at room temperature before conversion to Mg-ATP-actin and processing as for the control. Profilin: Profilin was added to Ca-ATP-G-actin at a 2:1 ratio. Control reactions lacking latrunculin or profilin contained G-actin (MA-L) that was converted to Mg-ATP-G-actin and allowed to polymerize to steady state conditions before dilution to the indicated concentrations. Chimera 2 of Tβ4 (Chim2-Tβ4) for a final concentration of 5 μM was added to muscle G-actin (MA-L) under conditions preventing salt-induced polymerization in G-buffer and preincubated for 10 min at 30 °C. None of the molecules tested affected the low basal ExoY activity in the absence of actin. Error bars correspond to s.d. of two experimental replicates.

We next tested whether purified actin could activate ExoY *in vitro.* We used buffer conditions allowing actin self-assembly to proceed above the critical concentration for polymerization. Highly pure non-muscle (cytoplasmic) actin isolated from human platelets (Cytoskeleton, Inc., designated here A-99) strongly stimulated the adenylate cyclase activity of ExoY (HF-ExoY), with a maximal activity reaching 120 μmol of cAMP min^−1^ mg^−1^ (Fig. 3a). Since expression of ExoY in transfected mammalian cells led to accumulation of cGMP to levels exceeding that of cAMP^7^,^8^, we also tested GTP as substrate. We found that the guanylate cyclase activity of HF-ExoY was ∼8 times higher than the adenylate cyclase activity in the presence of actin *in vitro* (Fig. 3a). The background activity without actin was estimated to be about 1 nmol min^−1^ mg^−1^ for cGMP and cAMP synthesis. Thus, the ExoY nucleotidyl cyclase activity was stimulated more than 10,000 fold by submicromolar concentrations of actin. Different mammalian actin isoforms (A-99 consisting of 85% β- and 15% γ-actin, or α-actin from rabbit skeletal muscle) were tested and all found to strongly activate ExoY-catalysed synthesis of cGMP in a concentration dependent manner (Supplementary Fig. 2), indicating that they were all effective activators of ExoY. Subsequent experiments were performed using α-actin from rabbit skeletal muscle purified in our laboratory and fully functional in actin polymerization assays (>95% pure, designated MA-L).

To examine a possible dependence of ExoY activation on the different states of actin (ATP- versus ADP-bound, monomeric versus polymeric forms), we measured cGMP synthesis activity of ExoY at different actin concentrations below or above the critical concentrations that favor actin self-assembly in these different nucleotide states. Measurements were performed at increasing concentrations of actin that was initially loaded with either Mg-ATP or Mg-ADP. A similar maximal activity of 1,000–1,200 μmol of cGMP min^−1^ mg^−1^ was obtained with both ATP- and ADP-bound actin (Fig. 3b). In contrast, the actin concentrations required for half maximal activation of ExoY (K_1/2_) were dependent on the bound nucleotides: half maximal ExoY activation was obtained using 0.2 μM of ATP-loaded actin (Fig. 3b), just above the critical concentration of 0.1 μM at which Mg-ATP-actin spontaneously polymerizes with salt. Conversely, half-maximal ExoY activation was obtained at about 2.4 μM ADP-loaded actin (Fig. 3b), a value close to the critical spontaneous polymerizing concentration (about 2 μM) of Mg-ADP-actin^22^. Altogether, these results suggest that the maximal activation of ExoY by actin was correlated with F-actin formation.

### Effect of latrunculin A and G-actin-binding proteins

We examined whether proteins or molecules known to bind to G-actin and to inhibit elongation and/or spontaneous nucleation, alter the activation of ExoY by actin. We used latrunculin A or the abundant G-actin-binding proteins profilin and thymosin-β4 (Tβ4). These three molecules are known to bind to distinct G-actin interfaces: (i) The small macrolide latrunculin A^23^,^24^ inhibits actin self-assembly by binding (*K*_d_∼0.2 μM) to a cleft located on the pointed face of G-actin. (ii) Profilin binds (*K*_d_∼0.1 μM) to the opposite face of monomers, called barbed face, and favors *in vivo* the unidirectional elongation of the most-dynamic barbed ends of filaments. *In vitro*, G-actin:profilin complexes inhibit actin spontaneous nucleation and thus polymerization in the absence of actin nuclei or filament seeds. (iii) Tβ4 acts as a major G-actin-sequestering polypeptide in cells^25^,^26^. Here we used a chimeric β-thymosin domain, chim2-Tβ4 (a chimera between bovine Tβ4 and Drosophila ciboulot made of β-thymosin repeats), which sequesters G-actin monomers with higher affinity than Tβ4 (*K*_d_∼0.5 μM versus 2 μM)^25^,^26^. As with Tβ4, the binding interface of chim2-Tβ4 with actin monomers is extensive, covering both the barbed and pointed faces^25^,^26^.

Actin monomers were saturated with these molecules to inhibit the spontaneous nucleation or polymerization of actin. As shown in Fig. 3c, latrunculin A, profilin and chim2-Tβ4, all efficiently inhibited ExoY activation by actin at concentrations that fully activate the enzyme in control assays. These data thus provide additional indications that filamentous actin is the preferred activator of ExoY.

### ExoY binding along actin filaments alters their turnover

**Figure 4:**
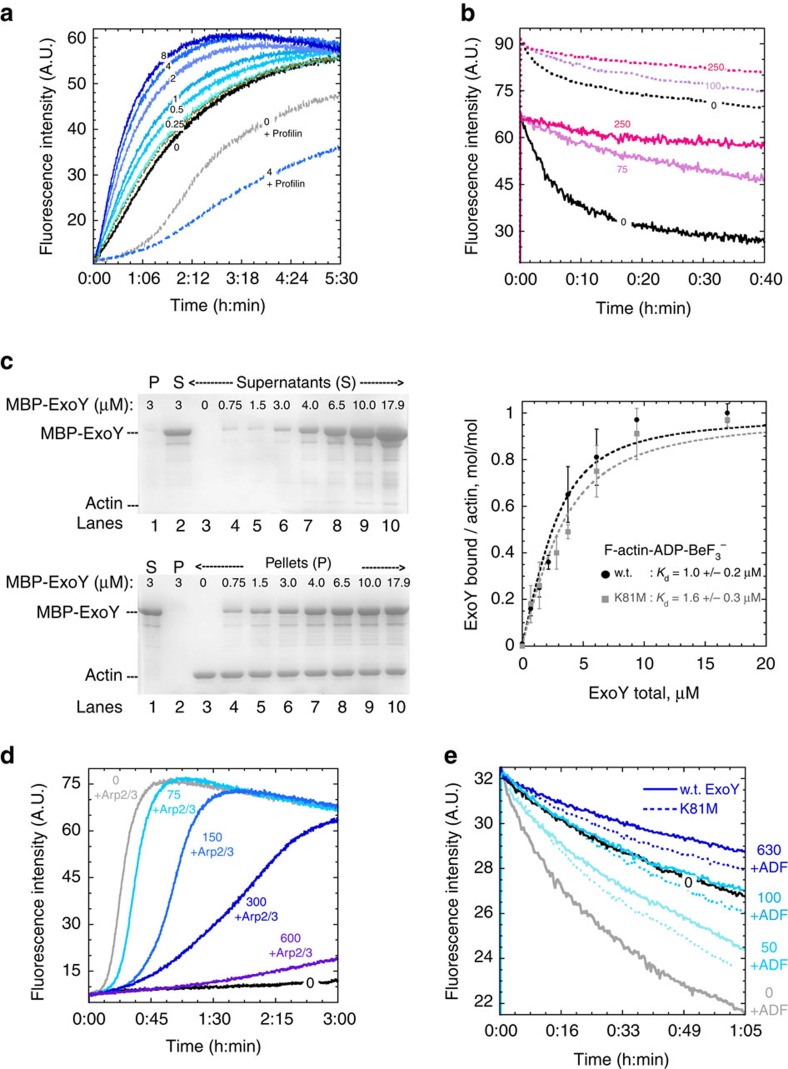
ExoY association with F-actin can interfere with the regulation of filament dynamics by eukaryotic cytoskeletal proteins. (**a**) MBP-ExoY^K81M^ mediated acceleration of G-actin-ATP-Mg self-assembly rate and its inhibition by profilin. Four micromolar G-actin-Mg-ATP (3% pyrenyl labelled) was polymerized in the absence (continuous lines) or presence (dashed lines) of 12 μM profilin at the indicated concentrations (μM) of MBP-ExoY^K81M^. MBP by itself (4 μM, brown) did not stimulate polymerization. (**b**) ExoY^K81M^ binding to filaments inhibits their spontaneous disassembly kinetics from free barbed- or pointed-ends. Disassembly from free barbed-ends (continuous lines): F-actin (2 μM, 50% pyrenyl) at steady state was diluted to 50 nM in the presence of 0 to 250 nM ExoY^K81M^. Disassembly from pointed-ends (dotted lines): filaments with their barbed-ends capped by Gelsolin (10 μM actin, 33.3 nM Gelsolin) at steady state were diluted to 300 nM in the presence of 0 to 250 nM ExoY^K81M^. (**c**) Binding affinity of MBP-fused ExoY and ExoY^K81M^ for F-actin (MA-L) measured in high-speed cosedimentation assays. Left panels: Representative Coomassie blue stained SDS–PAGE (10%) gel images from the supernatant (S) and pellet (P) fractions using 3 μM of F-actin-ADP-BeF_3_^−^ mixed with increasing amount of MBP-ExoY (0 to 17.9 μM). Without F-actin (lanes 1 and 2) no more than 7% of MBP-ExoY was found in the pellet. Right panel: The increasing amounts of MBP-ExoY/ExoY^K81M^ bound to filaments were measured by densitometry, normalized and fitted to derive the equilibrium dissociation constant (*K*_d_) of the proteins for F-actin-ADP-BeF_3_^−^. Error bars are s.e. (*n*≥3). (**d**) ExoY^K81M^ inhibits the acceleration of filament formation induced by VCA-activated Arp2/3 complex. Three micromolar G-actin-Mg-ATP (3% pyrenyl) was polymerized with 7.5 μM profilin, 0.2 μM NWASP-VCA, in the absence (black) or presence of 35 nM Arp2/3 and 0 to 600 nM ExoY^K81M^. (**e**) ExoY/ExoY^K81M^ inhibits the acceleration of F-actin-ADP disassembly promoted by ADF. F-actin-ADP (9 μM, 50% pyrenyl) at steady state was diluted to 4 μM and preincubated for 2 min with 0 to 630 nM ExoY (w.t., continuous lines) or ExoY^K81M^ (dotted lines) before depolymerization assays without ADF (0 nM ExoY/ExoY^K81M^, black) or with 4 μM ADF (grey, cyan or blue curves).

We examined whether ExoY interaction with actin alters the intrinsic or regulated dynamics of actin self-assembly *in vitro*. To avoid indirect effects of ATP depletion due to ExoY activity, we used the inactive variant ExoY^K81M^ in assembly*/*disassembly kinetic studies with G-/F-actin-ATP. Alternatively, wild-type ExoY was used in kinetic studies with G-/F-actin-ADP, since ADP is not an ExoY substrate. The kinetics of polymerization or depolymerization were monitored by following the increase or decrease, respectively, of pyrene-actin fluorescence intensity (pyrenyl-labelled actin subunits exhibit higher fluorescence when incorporated in filaments than free in solution). In polymerization assays, ExoY^K81M^ slightly accelerated the rate of G-actin-ATP-Mg or G-actin-ADP-Mg self-assembly (Fig. 4a, Supplementary Fig. 4), confirming that ExoY interacts with actin without preventing actin self-assembly. Yet, this stimulation of G-actin-ATP/ADP polymerization by ExoY was detected only at high ExoY concentrations (in μM range). Besides, ExoY-stimulated actin polymerization was abolished when G-actin was saturated by profilin (Fig. 4a). In eukaryotic cells, the polymerization competent G-actin pool is mainly bound to profilin^22^,^27^. Therefore, these results indicate that ExoY is unlikely to stimulate actin polymerization in host cells. To delineate the interaction of ExoY with F-actin, we performed filament disassembly assays monitored from free barbed- or pointed-ends. As shown in Fig. 4b, ExoY^K81M^ inhibited spontaneous F-actin disassembly induced by dilution. This inhibition was also observed when barbed ends were capped by gelsolin (Fig. 4b), thus excluding the possibility that ExoY inhibited disassembly by binding to barbed ends. These results, as well as the absence of ExoY effects on pointed and barbed-end elongation rates (Supplementary Fig. 5), indicate that ExoY binds along the sides of filaments. ExoY thus stabilizes actin inter-subunit contacts by interacting with adjacent subunits and prevents spontaneous disassembly of filaments.

The binding of ExoY and ExoY^K81M^ to filamentous actin was quantified by cosedimentation assays using F-actin steadily polymerized in the presence of ADP-BeF_3_^−^ (mimicking the ADP-Pi state of filaments) in order to keep most actin firmly polymerized despite the efficient conversion of ATP into cAMP by activated ExoY. Because ExoY and actin (43 and 42 kDa, respectively) cannot be well separated by SDS–PAGE, we used an ExoY protein fused to the maltose-binding-protein (MBP), MBP-ExoY. Cosedimentation assays were performed at constant concentration of F-actin using increasing concentrations of MBP-ExoY/ExoY^K81M^. As shown in Fig. 4c, MBP-ExoY or MBP-ExoY^K81M^ alone partitioned into the supernatant (lanes 1 and 2). The proteins that cosedimented with F-actin gradually increased with increasing MBP-ExoY/ExoY^K81M^ concentrations up to a saturation corresponding to an ExoY:actin stoichiometry of 1:1 (Fig. 4c, right panel). The estimated dissociation constants (*K*_d_) of MBP-ExoY and MBP-ExoY^K81M^ for F-actin-ADP-BeF_3_^−^ (1.0±0.2 μM and 1.6±0.3 μM, respectively) or MBP-ExoY^K81M^ for F-actin (1.6±0.4 μM) (Supplementary Fig. 6a), were in the same range as those of many eukaryotic cytoskeletal proteins that bind along filaments^22^,^27^,^28^,^29^.

Finally, we analysed whether ExoY binding to F-actin could interfere with the regulation of filament dynamics by eukaryotic cytoskeletal proteins known to bind along the sides of filaments. We considered two key regulatory proteins, which are ubiquitous among eukaryotic cells: the Arp2/3 complex and actin-depolymerizing factor (ADF). The Arp2/3 complex, upon its activation by VCA domains of the WASP family proteins, binds to the side of a pre-existing filament where it catalyses actin filament branching^22^,^27^,^30^. ADF/cofilin proteins, present at micromolar concentrations in eukaryotic cells, bind cooperatively and preferentially to F-actin-ADP subunits along filaments (*K*_d_∼0.1 μM), severing aged filaments, and enhancing their disassembly and turnover^22^,^27^,^31^.

In experiments with the Arp2/3 complex, we performed actin polymerization assays using G-actin saturated by profilin to approach a more physiological context. As shown in Fig. 4d, the acceleration of G-actin-ATP polymerization by VCA-activated Arp2/3 (25–35 nM) was inhibited by ExoY^K81M^ at concentrations of 100 nM and higher. This demonstrates that ExoY antagonizes the binding of the activated Arp2/3 complex along filaments and hence VCA-Arp2/3 regulation.

In dilution-induced F-actin-ADP disassembly assays (Fig. 4e), ExoY or ExoY^K81M^ (100 nM) were able to completely inhibit the acceleration of filament disassembly promoted by ADF (4 μM) at a low ExoY:ADF ratio of 1:40. The sub-stoichiometric ratio of ExoY with respect to ADF and the respective *K*_d_ for F-actin binding suggested that the complete inhibition of ADF activity by ExoY was not achieved by a direct competition for binding to F-actin. To investigate how ExoY inhibits the disassembly of F-actin mediated by ADF, we examined whether ExoY binding to F-actin imposes cooperative conformational perturbations on the F-actin structure and thus stabilizes filaments in a conformation incompatible with ADF binding and severing activity. We used the drug phalloidin, which binds with high affinity to F-actin. Compared with native filaments, phalloidin-stabilized filaments appear stiffer and exhibit an altered conformation^32^ that inhibits the cooperative binding and activity of ADF. To test whether ExoY imposes cooperative conformational perturbations on F-actin, we measured the binding affinity of ExoY^K81M^ to phalloidin-modified filaments using cosedimentation assays. For three different conformational states of F-actin: native, phalloidin-bound (Supplementary Fig. 6a), or F-actin-ADP-BeF_3_^−^ (Fig. 4c), the affinity of MBP-ExoY^K81M^ for filaments remained unchanged (*K*_d_∼1.6–1.8 μM). Thus, ExoY binding does not appear to induce a particular cooperative conformational change in F-actin. Then we examined whether a pre-incubation of actin filaments with MBP-ExoY^K81M^ altered subsequent binding of ADF to F-actin. We used the low MBP-ExoY^K81M^:ADF ratios that antagonized ADF activity in the depolymerization assays of Fig. 4e. We found no significant change of ADF binding to F-actin at a low MBP-ExoY^K81M^:ADF ratio of 1:40 and a slight decrease (about 16%) at a 1:6.7 ratio (Supplementary Fig. 6b). This suggests that ExoY binding to F-actin antagonizes ADF depolymerizing activity by preventing its cooperative binding along actin-ADP filaments.

### F-actin cell content increases upon ExoY recruitment to cables

**Figure 5:**
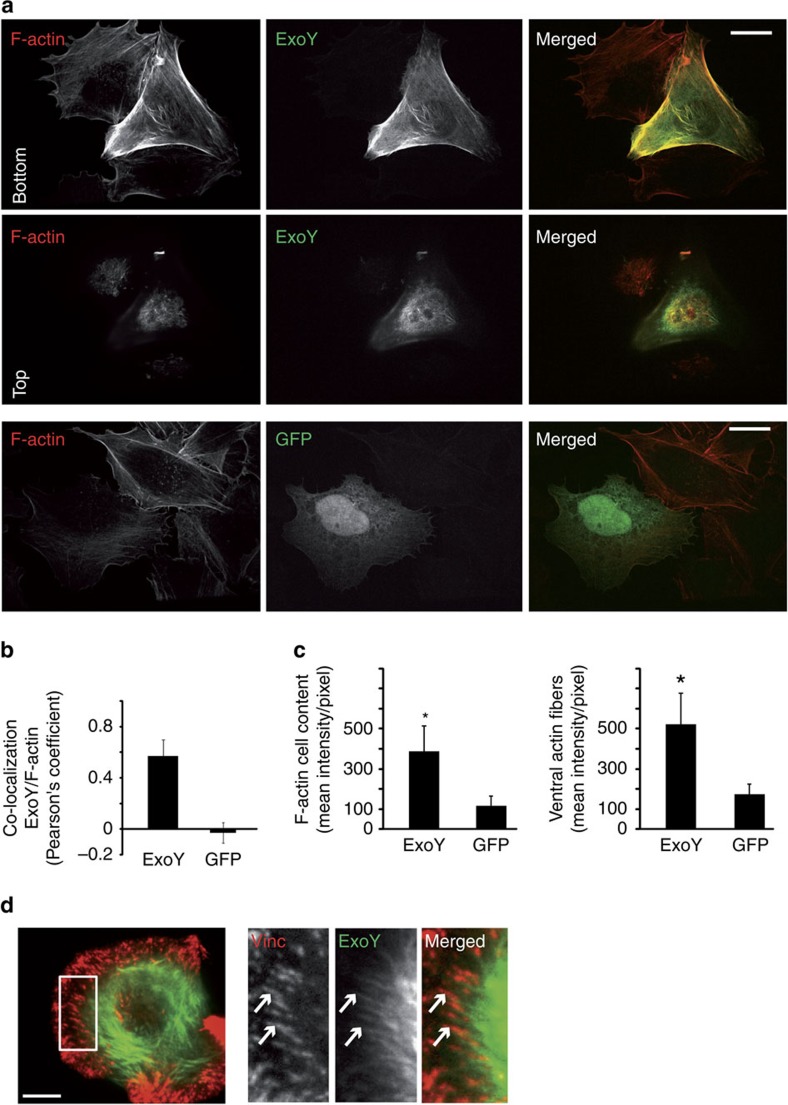
ExoY co-localizes with actin cables and increases F-actin cell content. (**a**) Staining of F-actin (670 nm fluorescent phalloidin) in HeLa cells transfected with ExoY^K81M^-AcGFP construct. Scale bars, 10 μm. Spinning disk confocal microscopy pictures show co-localization of ExoY^K81M^-AcGFP with F-actin from the bottom slices or the top ones and increased phalloidin staining in comparison to the control (AcGFP). (**b**) The co-localization of ExoY^K81M^-AcGFP with F-actin was estimated by measuring the average Pearson's correlation coefficient (±s.d.; *n*≥15 cells) between AcGFP and 670 nm fluorescence signals in individual whole cells (without extracellular regions). (**c**) Quantification of F-actin within whole cells (from bottom to top slices in each cell) and of actin stress fibres at the bottom slices in ExoY^K81M^-AcGFP- or AcGFP-expressing cells. Data are mean fluorescent intensity per pixel ±s.d. (*n*≥15 cells; *P<0.001; two tail Student's *t*-test). (**d**) TIRF pictures of HeLa cell expressing ExoY^K81M^-AcGFP and mCherry-vinculin. Scale bars, 5 μm. ExoY^K81M^-AcGFP-labelled stress fibres terminated at vinculin-labelled focal adhesions (arrows).

To our knowledge, the localization of ExoY in eukaryotic cells was not previously reported and is of particular interest in light of its direct interaction with F-actin *in vitro*. To avoid toxic effects of ExoY when expressed in eukaryotic cells, the catalytically inactive variant ExoY^K81M^ fused to AcGFP (a monomeric green fluorescent protein) was expressed in transiently transfected HeLa cells (a human cell line of epithelial origin). Co-localization of ExoY^K81M^-AcGFP with F-actin was first analysed using spinning disk confocal microscopy of phalloidin-stained cells. Phalloidin was conjugated to a far-red-670 nm fluorophore to ensure signal separation from AcGFP. We observed the recruitment of ExoY to F-actin rich structures in particular along the plasma membrane, to ruffles and to actin cables (Fig. 5a). Calculated Pearson's correlation coefficient (PCC) of 0.57±0.15 (mean±s.d.; n>15 cells) showed significant co-localization of ExoY^K81M^-AcGFP with F-actin, whereas the transfection of AcGFP alone led to a negative PCC (Fig. 5b). To show ExoY co-localization with actin fibres, we next performed cotransfection with mCherry-Vinculin, a protein of focal adhesions. Contractile actin fibres are often connected to focal adhesion at their ends. Using total internal reflection fluorescence (TIRF) microscopy, we observed that ExoY^K81M^-AcGFP labelled stress fibres especially at vinculin labelled focal adhesion (Fig. 5d), whereas GFP alone was uniformly distributed (Supplementary Fig. 7). Quantifications of phalloidin staining of spinning disk confocal microscopy pictures indicated additionally that F-actin content was increased in ExoY-expressing cells. The mean fluorescent intensities of F-actin over all section surfaces (from bottom to top within each cell), or of actin stress fibres measured at the bottom slices, were higher than in control AcGFP-expressing cells (Fig. 5c). In agreement with our biochemical analysis of ExoY in F-actin disassembly assays, these data establish that the bacterial toxin can bind to and stabilize microfilaments in host cells in the presence of myriad actin-binding regulatory proteins.

### Activation of a *Vibrio* ExoY-like protein by actin

**Figure 6:**
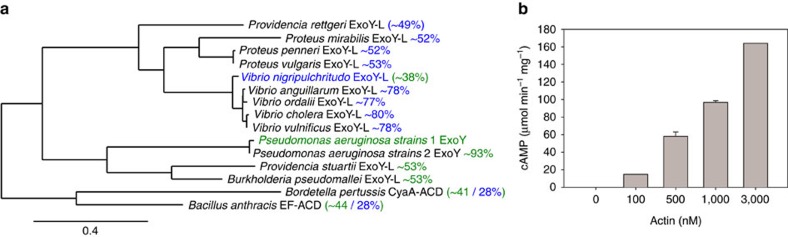
The bacterial ExoY-like nucleotidyl cyclase toxin subfamily. (**a**) Phylogenetic tree. The amino-acid sequences of *Pseudomonas aeruginosa* ExoY and various ExoY-related effector domains/toxins found in several emerging Gram-negative bacterial pathogens were aligned as shown in Supplementary Fig. 8 and clustered on phylogram branches on the basis of the similarity of their amino-acid sequences using the Phylogeny.fr platform^61^. Calmodulin-activated edema factor and Cya Adenylate Cyclase Domains (ACD) were used as an outgroup more distantly related to ExoY-like nucleotidyl cyclase toxins to root the phylogeny. NCBI accessions of bacterial protein sequences are given in Supplementary Table 2. Pairwise sequence similarities (%) with *P. aeruginosa* ExoY or *V. nigripulchritudo* ExoY-like (VnExoY-L) are given in green and blue, respectively (see Supplementary Table 2 for more details). Similarity values without parenthesis indicate the ExoY-like sequences that are the most significantly related to actin-activated ExoY or VnExoY-L nucleotidyl cyclases. (**b**) Activation of VnExoY-L catalysed synthesis of cAMP by actin (MA-L). Reactions containing 10 ng VnExoY-L (3.7 nM) and actin at indicated concentrations were started by the addition of 2 mM ATP and incubated for 30 min at 30 °C. The background activity without actin was about 1 nmol of cAMP min^−1^ mg^−1^. Error bars correspond to s.d. of two experimental replicates.

We further examined whether actin could activate other putative ExoY-like cyclases that are present in a number of MARTX toxins from pathogenic proteobacterial species present in the genera *Burkholderia*, *Vibrio*^16^, *Providencia* or *Proteus* (Fig. 6a). For this, we selected the ExoY-like module from the MARTX toxin encoded by the virulence-associated plasmid pA_SFn1_ from *V. nigripulchritudo*^16^,^33^, an emerging marine pathogen infecting farmed shrimps. The multidomain MARTX toxin is processed inside host cells into individual effector domains by an inbuilt cysteine protein domain^34^. The N- and C-terminus of the *V. nigripulchritudo* ExoY-like effector domain were chosen based on sequence alignments of *P. aeruginosa* ExoY and ExoY-like modules from several *Vibrio* MARTX toxins (Supplementary Fig. 8), the signature of cysteine protein domain cleavage sites present in some *Vibrio* ExoY-like modules and hydrophobic cluster analysis secondary structure prediction analysis. The MARTX-ExoY-like protein of 53 kDa corresponding to residues Y3412 to L3872 of Uniprot reference F0V1C5 was termed here VnExoY-L. The protein carrying a C-terminal Flag-His tag (VnExoY-L-FH) was purified and tested for its adenylate cyclase activity in the presence and absence of actin. Figure 6b shows that VnExoY-L displayed a potent adenylate cyclase activity in the presence of actin, which stimulates the enzymatic activity more than 10,000 fold. In contrast to *P. aeruginosa* ExoY, VnExoY-L did not exhibit any cGMP synthesizing activity. We conclude that actin may be a common activator of the various ExoY-like cyclase modules.

### Identification of actin mutants that fail to activate ExoY

**Figure 7:**
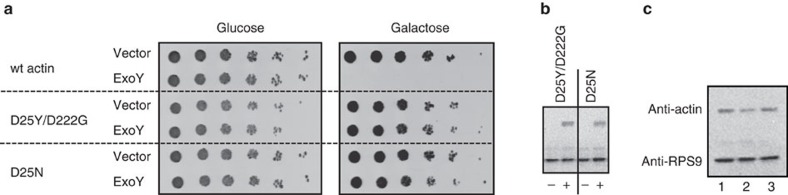
Actin mutant alleles affecting the activation of ExoY. (**a**) Drop tests of serial dilutions of *S. cerevisiae* strains expressing wild-type (wt) actin (SC489), D25Y/D222G (SC690) or D25N (SC691) and p1593 for galactose-induced expression of ExoY or the corresponding vector control YEpGal555. Cell suspensions were normalized to an OD600 of 1.0 and 5-fold serial dilutions were applied as 3 μl drops on SD or SG-agar plates. (**b**) Western blot analysis to verify expression of Myc-ExoY in SC690 and SC691 with anti-Myc or anti-RPS9 (loading control). (**c**) Western blot analysis to verify expression of actin in SC489 (1), SC690 (2) and SC691 (3) with anti-actin or anti-RPS9 (loading control). Uncropped images of (**b**,**c**) are shown in Supplementary Fig. 10.

To further delineate the molecular mechanism of activation of ExoY by actin, we attempted to identify actin mutants with impaired ability to activate ExoY. For this purpose, we used a recently developed yeast genetic screen, in which the wild-type actin of *S. cerevisiae* is replaced by actin variants expressed from a plasmid^35^. *S. cerevisiae* contains a single actin gene (*act1*), which can be deleted provided the strain harbours a plasmid (with an URA3 marker) carrying a complementary copy of wild-type actin (*S. cerevisiae act1*::LEU2+pACT1 [URA3]). This recombinant strain was then transformed with a pool of plasmids carrying the HIS3 auxotrophic marker and expressing actin variants generated by *in vitro* mutagenesis (see Methods). Subsequently, the URA3-containing plasmid was eliminated (by selection on 5-fluoroorotic acid containing medium)^36^ and the resulting cells, therefore, expressed only the actin variants encoded by the HIS3-plasmid^35^. These cells were transformed with a plasmid (p1654) expressing ExoY (Myc-ExoY-NanoLuc) under the control of the galactose-inducible GAL1 promoter and cells that tolerated ExoY expression were isolated. Twenty-one ExoY-resistant colonies (with unimpaired ExoY expression levels) were selected and found to harbour two different actin mutant alleles: 14 mutants had a double substitution, D25Y and D222G, and 7 had a single modification D25N. Figure 7a compares growth of *S. cerevisiae* strains expressing wild-type actin (SC489), mutant D25Y/D222G (SC690) or mutant D25N (SC691) and ExoY in drop tests of sequentially diluted cultures. Whereas ExoY expression (that is, in the presence of galactose) totally inhibited the growth of the wild-type strain, it did not affect the growth of the yeast cells harbouring either one of the two actin mutants. We verified ExoY expression in the actin mutant strains by western blots with anti-Myc antibodies (Fig. 7b). Both actin variants were similarly expressed (Fig. 7c) and supported growth of *S. cerevisiae*: the double mutant grew slightly more slowly in SD minimal medium as compared with the wild type and the single mutant (doubling time 3 h versus 2.5 h, respectively) and was slightly affected under different stress conditions tested: at 15 or 37 °C or in the presence of 1M NaCl (Supplementary Fig. 9). Crude extracts of the different *S. cerevisiae* strains were then prepared and tested for their ability to activate ExoY *in vitro*. While extracts from wild-type cells strongly stimulated ExoY, no activity of ExoY was detected in extracts from yeasts expressing the actin mutants (Table 1). In addition, we found that the actin mutations D25Y/D222G or D25N also largely impaired the activation of the *Vibrio* enzyme Vn-ExoY *in vitro* (Table 1).

## Discussion

Bacterial toxins that use substrates not unique to eukaryotic organisms need to be kept inactive within the bacterial pathogen in order to prevent detrimental effects to the native host. Once they enter the eukaryotic host cell, the toxin is usually activated by a host cell cofactor that typically represents a specific and abundant protein/marker of their hosts. Actin fits these criteria perfectly well: actin is absent from bacteria, found in essentially all eukaryotic cells where it is one of the most abundant proteins, and despite evolutionary separation by billions of years, *S. cerevisiae* and human actin share 87% amino-acid sequence identity. These features make actin also a frequent target of bacterial toxins that can affect the polymerization state of actin in different ways by introducing modifications, such as ADP-ribosylation at different position or crosslinking (for reviews see refs 37, 38). The provoked rearrangements have a profound effect on the cytoskeleton of the host cells and affect their response to bacterial invasion.

Here we show that actin is a potent activator of a group of bacterial toxins that are homologous to the *P. aeruginosa* ExoY effector and that display nucleotidyl cyclase activities with different substrate selectivity.

We demonstrate that mammalian actin is able to stimulate ExoY's adenylate and guanylate cyclase activities more than 10,000 fold to reach specific activities (in optimum conditions) of about 120 and 900 μmol min^−1^ mg^−1^ for cAMP and cGMP synthesis, respectively (Fig. 3a). The higher guanylate cyclase activity as compared with the adenylate cyclase one is in agreement with the preferential accumulation of cGMP over cAMP observed *in vivo*^7^,^8^. The corresponding kcat for cGMP synthesis is approaching 1,000 s^−1^ and, therefore, within the same order of magnitude as the catalytic rates measured for cAMP synthesis for the related cyclase toxins CyaA or edema factor, when activated by calmodulin, their common eukaryotic activator^39^,^40^.

Using a yeast genetic screen (Fig. 7), we further identified an actin mutation (D25N) that blunts actin's ability to activate ExoY. D25 in actin subdomain 1 is a charged residue solvent-exposed in F-actin and can thus be part of the contact interface of F-actin-binding proteins^41^. These data not only confirm that actin is an activator of ExoY but demonstrate that actin is the only activator of ExoY present in yeast. We can thus exclude the possibility that a contaminant present in the actin preparations could be responsible for activation of ExoY in our *in vitro* assays.

Our results support the view that polymerization of G-actin into F-actin is critical for maximum activation of ExoY. First, the maximal activation of ExoY by actin-ATP or actin-ADP was correlated with F-actin formation in each nucleotide state (Fig. 3b). Second, ExoY activation by actin was strongly antagonized by different G-actin-binding proteins, such as profilin, or a Tβ4-derivative protein with similar sequestering activity as Tβ4 (chim2-Tβ4), or by latrunculin A that prevents actin polymerization (Fig. 3c). Finally, ExoY binds along naked filaments with a low micromolar affinity (*K*_d_ of about 1±0.2 μM, Fig. 4c), which should allow efficient competition *in vivo* with many eukaryotic cytoskeletal side-binding proteins that also bind along filaments with low micromolar affinity^27^,^28^,^29^. The co-localization of ExoY-GFP with F-actin rich structures confirmed that ExoY indeed associates with actin filaments within cells (Fig. 5).

ExoY thus represents to our knowledge the first example of a bacterial toxin that is activated by F-actin. G-actin has been shown previously to activate a bacterial toxin secreted by the T3SS namely YopO/YpkA, a multidomain protein from *Yersinia* species (Y*. enterocolitica* and *Y. pseudotuberculosis*, respectively), which is involved in the disruption of the actin cytoskeleton^42^,^43^. In contrast, *P. aeruginosa* ExoY binding to F-actin inhibits the spontaneous or regulated dynamics of F-actin disassembly *in vitro* (Fig. 4b,e) increasing the microfilament content in transfected cells (Fig. 5). YopO binding with an actin monomer induces autophosphorylation and activation of its N-terminal serine/threonine kinase domain^43^. In the YopO:G–actin complex, the bound actin is sequestered from polymerization and used subsequently as bait to recruit, phosphorylate and thus misregulate various host actin-regulating proteins^44^. The mechanisms of activation of ExoY and YopO by F- and G-actin, respectively, are, therefore, likely different.

While most of the ExoY related toxicity in infected cells depends on its nucleotidyl cyclase activity, its direct binding to actin filaments could additionally alter normal host cell homeostasis. We showed that *in vitro* (Fig. 4e), ExoY (as low as 50 nM) can antagonize the cooperative activity of ADF at sub-molar ratios of ExoY with respect to the regulatory cytoskeletal side-binding protein. Even though ExoY may be present at lower concentrations in host cells, its binding along actin filaments might nevertheless contribute to destabilizing precise spatial and temporal regulation of actin dynamics in eukaryotic cells. Huber *et al*.^15^ have recently observed that at 3 h post-infection, a *P. aeruginosa* T3SS effector mutant strain expressing only ExoY showed slightly increased human endothelial cell spreading, suggesting a stabilization of the actin cytoskeleton. It will thus be interesting to examine in more details, the actin cytoskeleton dynamics of host cells upon infection with bacteria expressing catalytically inactive ExoY or ExoY-like proteins alone or together with other toxins affecting actin cytoskeleton regulation (ExoS, ExoT from *P. aeruginosa,* actin cross-linking (ACD) or Rho-GTPase inactivation domain (RID) from various MARTX toxins of the *Vibrio* genus^16^). Given that ExoS and ExoT disrupt actin filaments, cytotoxicity of ExoY might be self-limited by the interplay between *P. aeruginosa* T3SS toxins. Such an interplay between ExoY and ExoS or ExoT activities via the actin cytoskeleton integrity may explain why a mutant strain of *P. aeruginosa* injecting only ExoY as T3SS effector was found more potent for inducing high cAMP levels in infected human endothelial cells than the wild-type or mutant strains expressing all three T3SS toxins or ExoY with either ExoS or ExoT, respectively^15^.

ExoY-like modules are frequently found among the effector domains of MARTX toxins in multiple bacterial species of the *Vibrio* genus^16^, which represent emerging human or animal pathogens. In addition, ExoY-like proteins can be found in various other Gram-negative pathogenic bacteria from the genus *Providencia, Burkholderia* or *Proteus* (Fig. 6a). Here we showed that VnExoY-L, a rather distantly related ExoY-like module from *V. nigripulchritudo*, was also strongly stimulated (more than 10,000 fold) by actin and efficiently synthesized cAMP but not cGMP (Fig. 6b). The lack of guanylate cyclase activity is in agreement with the results obtained with the *V. vulnificus* ExoY-like module^16^, a close homologue of VnExoY-L (>75% sequence similarity, Fig. 6a and Supplementary Table 2), and may thus reflect a more general difference regarding the nucleotide substrate specificities between the *P. aeruginosa* ExoY and other ExoY-like proteins found in MARTX toxins similar to those present in the *Vibrio* genus (Fig. 6a and Supplementary Fig. 8).

Actin thus represents a common eukaryotic activator for several exoenzymes (Fig. 6a) within the class II adenylyl cyclase toxin family (described in ref. 19). We propose the term actin-activated nucleotidyl cyclase toxins to describe this particular sub-family.

## Methods

### Strains and growth conditions

Strains, plasmids and primers are described in Supplementary table 1.

*E. coli* strains were grown in lysogeny broth. Ampicillin (100 μg ml^−1^) was added for plasmid maintenance in *E. coli*. *S. cerevisiae* strains were grown in yeast extract peptone dextrose or yeast extract peptone galactose media or in minimal medium containing yeast nitrogene base without amino acids (Difco) containing galactose (SG) or glucose (SD) supplemented with uracil, histidine, tryptophane and/or adenine if required. Glucose or galactose was present at 2%. Hygromycin (Sigma) was present at 200 μg ml^−1^, to maintain plasmids in yeast. *S. cerevisiae* strains were transformed using the lithium-acetate method^45^.

The plasmid for expression of HF-ExoY under control of the arabinose-inducible promoter P_*ara*_(pUM447) was cloned as follows: Primers UM248 and UM254 were used to PCR-amplify *exoY* from *P. aeruginosa* PAO1 chromosomal DNA. The EcoRI, XbaI digested fragment was used to replace the EcoRI/XbaI fragment of pUM407. pUM449 for arabinose-inducible expression of ExoY-FH was constructed by PCR-amplifying *exoY* using UM245 and UM250, digestion with EcoRI and XhoI and replacing the EcoRI/XhoI fragment from pUM407. Protein expression levels were low. We, therefore, constructed a plasmid expressing ExoY-FH from λP_*L*_ controlled by the temperature sensitive cI repressor (cI857), pUM460. For this, the fragment coding for ExoY-FH from pUM449, was PCR-amplified using primer UM254 and UM255 and cloned as NcoI/XbaI fragment into the same sites of plasmid pTRCAG.

pUM483 and pUM482 for expression of TAP-tagged or HA-tagged ExoY, respectively in *S. cerevisiae* were cloned using Gateway technology. The entry vector (pUM478) was created in pDONR221 (Invitrogen) by introducing a PCR fragment amplified with primers UM282 and 283 from template pUM449. The fragment coding for ExoY was transferred by *in vitro* recombination from pUM478 into to the destination vectors pAG415GAL-ccdB-TAP and pAG415GAL-ccdB-HA (ref. 46). The LEU2 marker was then exchanged to a hygromycin resistance cassette by *in vivo* recombination as follows: The 6.8 or 7.6 kb fragment obtained after MunI digestion of pUM483 or pUM482, respectively was isolated from agarose gel and transformed into *S. cerevisiae* together with the hygromycin resistance cassette which was PCR amplified from pAG32 using primer UM302 and UM303 to yield pUM484 (ExoY-TAP, Hygromycin^R^) and pUM485 (ExoY-HA, Hygromycin^R^), respectively.

For the construction of clones expressing the ExoY^K81M^ mutant, two PCR fragments were amplified using pUM451 as template: PCR1 was performed with primers UM316 and UM318, and PCR2 was performed with primers UM319 and UM317. The outside primers UM316 and UM317 and equimolar amounts of PCR fragments 1 and 2 were used to perform overlapping PCR. The obtained PCR fragment was directly placed into pGEM-T Easy (Promega) by TA cloning. A sequence verified clone (pUM492) was used as source for the 155 bp SphI/SalI fragment containing the mutated sequence part of *exoY* to replace the SphI/SalI fragment from pUM484 and pUM485 yielding pUM497 (ExoY^K81M^-TAP) and pUM498 (ExoY^K81M^-HA), respectively.

pUM503 expressing ExoY^K81M^-FH under λP_*L*_ -cI857 was created by exchanging the SacII/SphI fragment from pUM460 by that of pUM497.

pUM518 for the expression of ExoY^K81M^ fused to the N-terminus of AcGFP in mammalian cells was created by insertion of a NheI/XhoI digested PCR fragment that was amplified from pUM503 using primer UM345 and UM246 into pAcGFP-N1.

pUM522 for the expression of Vn-ExoY-L-FH from λP_*L*_ controlled by the temperature sensitive cI repressor (cI857) was constructed by replacing the NcoI/KpnI fragment expressing ExoY from pUM460 by the PCR fragment that was amplified from *V. nigripulchritudo* DNA using primer UM355 and UM356.

pEA11 and pEA12 for the expression of His-MBP-ExoY-ST or His-MBP-ExoY^K81M^-ST under control of the P_*tac*_ promoter were constructed as follows: Primers UM350 and UM246 were used to PCR-amplify *exoY* or the mutant gene from pUM445 or pUM502, respectively. The BglII, XhoI digested fragments were inserted into a modified pGEX-6-P1 (ref. 47) digested with BamHI and XhoI.

p1593 for the expression of ExoY with a N-terminal Myc-tag in yeast was created by insertion of a XhoI/KpnI digested PCR fragment that was amplified from pUM485 using primer 1259 and 1260 into YEpGal555 (ref. 35). ExoY expressed from p1595 was C-terminally fused to NanoLuciferase to allow quantification of expression. The fragment coding for NanoLuc was PCR-amplified from pNL1.1 (Promega) using primers 1261 and 1262 and fused to the *exoY* gene in p1593 using the KpnI and NheI sites.

p1559 was created by replacing the cassette coding for β-lactamase (Bla) in YEpGal555 by the kanamycin resistance cassette (KmR) from pVK-3. For this purpose a NcoI site was created 5′ of the *bla* gene of YEpGal555 by site-directed mutagenesis using primers 1217 and 1218. The NcoI/AhdI digested vector was blunted and ligated with the PstI digested and blunt ended fragment from pVK-3 coding for kmR. The SacI/NheI fragment from p1595 coding for ExoY was then inserted into this vector to yield p1654.

Plasmid p1387 was used for random mutagenesis of *S. cerevisiae act1* and was created from p1182 by removing the ClaI site located within *act1* to construct a plasmid with unique ClaI and SalI sites flanking the gene's second exon coding for amino acid 4 to the C'end of Act1.

### Protein purification

ExoY-FH and VnExoY-L-FH were purified by nickel affinity chromatography under denaturing conditions (in the presence of 8 M urea) from the non-soluble protein fraction obtained from 1 liter cultures of *E. coli* BLR (pUM460) or (pUM522), respectively. Proteins were expressed from the λP_*L*_ promoter controlled by the temperature sensitive cI repressor (cI857), which was induced by shifting the temperature from 30 to 42 °C. Proteins were renatured by dialysis into 25 mM Tris-HCl pH 9.0, 500 mM NaCl, 10% glycerol, 1 mM 1,4-dithiothreitol (DTT) for ExoY-FH and 50 mM Tris-HCl pH 8.0, 200 mM NaCl, 10% glycerol, 1 mM DTT for VnExoY-FH. HF-ExoY was purified from the soluble fraction obtained from 0.5 l cultures of MG1655 (pUM447) that were grown at 30 °C.

The fusion constructs of ExoY/ExoY^K81M^ with an N-terminal maltose-binding protein (MBP), designed as follows: (His-tag)-(MBP)-(PreScission-site)-(ExoY/ExoY^K81M^)-(Strep-tagII) and referred in the text as MBP-ExoY/ExoY^K81M^-ST, or their truncated forms (ExoY/ExoY^K81M^-ST) were purified under non-denaturing conditions successively from HisTrap, StrepTrap, and Superdex 200 16*60 columns using standard protocols. The His-tag-MBP fusion was either cleaved or not using PreScission protease before the StrepTrap purification step. Proteins were stored in 25 mM Tris-HCl pH 8.8, 150 mM KCl, 3 mM (NH_4_)_2_SO_4_, 0.5% Glycerol, 1 mM DTT. His-tagged MBP by itself had no effect alone on actin polymerization (Fig. 4a) or depolymerization rates.

Rabbit skeletal muscle alpha-actin was purified in our laboratory (referred as MA-L) using several cycles of polymerization and depolymerization as previously described^48^ and stored in G-buffer (5 mM Tris-HCl pH 7.8, 0.1 mM CaCl_2_, 0.2 mM ATP, 1 mM DTT). The purity of MA-L was estimated to be >95% according to analysis on denaturating SDS–PAGE gels. Functionality was controlled by several cycles of polymerization/depolymerization and by verifying that the measured actin concentrations of our samples fit the known critical concentration values of a fully functional and highly pure actin. ADP-actin was prepared by treatment of ATP-G-actin with hexokinase and glucose^49^.

Recombinant profilin I from mouse, chimera 2 of Thymosin-β4 and Drosophila ciboulot first β-Thymosin domain (chim2-Tβ4 referred as CH2 in ref. 25), full-length human gelsolin, VCA domain of human neural Wiskott-Aldrich syndrome protein (N-WASP), the Arp2/3 complex from bovine brain or Spectrin-actin seeds from human erythrocytes were purified as described previously^25^,^40^,^51^,^52^.

### Affinity purification

*S. cerevisiae* cells expressing ExoY^K81M^-TAP [TAP-tag as follows: Calmodulin-binding peptide separated by TEV protease cleavage site from the IgG-binding domain (amino acids 80–312 of protein A from *Staphylococcus aureus*, P02976] from pUM497 or ExoY^K81M^-HA [3 tandem HA (Human influenza hemagglutinin) tags] from pUM498 as mock control were grown in 2 L yeast extract peptone galactose at 30 °C. One step purification using Dynabeads M-270 Epoxy (Invitrogen) conjugated to rabbit IgG (Sigma, Ref. I5006) were performed according to ref. 53. One half of the methanol/chloroform precipitated protein was analysed by PAGE followed by staining with Bio-Safe Coomassie G-250 (BIO-RAD) followed by silver staining (Pierce). The second half was directly digested by trypsin and analysed by LC–MS/MS analysis at the proteomic facility of the Paris Descartes University (3P5) according to the details specified below. The raw data were analysed by MaxQuant 1.3.0.5. software (ref. 54) for protein identification and quantitative estimation of the specific enrichment of proteins in the experimental sample as compared with the control.

### LC–MS/MS analysis

Proteomics analyses were realized at the 3P5 proteomics facility, Université Paris Descartes, Sorbonne Paris Cité, Institut Cochin, Paris as previously described^55^. Briefly: LC–MS protein analysis: peptides from Trypsin-digested extracts were concentrated, washed and analysed using a reverse phase C18 column on an u3000 nanoHPLC hyphenated to a Linear Trap Quadrupole-Orbitrap mass spectrometer (all from Thermo). LTQ MS/MS CID spectra were acquired from up to 20 most abundant ions detected in the Orbitrap MS scan. Proteome discoverer 1.3 (Thermo) with Mascot (matrixscience^56^) was used for protein identification. Separate analyses were compared using the MyPROMS software^57^.

### Pull-downs and cosedimentation assays

Actin (MA-L) was converted to Mg-ATP actin and allowed to polymerize to steady state at 20 μM for 2 h at room temperature (Fig. 2a) or prevented from polymerization by the addition of latrunculin A to 30 μM (Fig. 2b), then diluted fivefold in binding buffer (50 mM Na-phosphate pH 8.0, 300 mM NaCl, 25 mM imidazole, 5 mM ATP, 20 mM MgCl_2_, complete EDTA-free protease inhibitor cocktail (Roche)) and added to 7.5 μl Ni-NTA agarose beads. Equimolar amounts (12 μg) of ExoY-FH or the corresponding buffer were added and binding was allowed in batch for 1 h at 4 °C rotating in Durapore filter units (Millipore). Samples were washed three times with 350 μl wash buffer 20 (as binding buffer but containing 20 mM imidazole and 0.5 mM ATP, and 6 μM latrunculin A for samples containing G-actin) and once in wash buffer 40 (as wash buffer 20 but containing 40 mM imidazole). Elution of bound proteins was performed by adding a 55 μl aliquot of hot (95 °C) SDS sample buffer (NuPage, Invitrogen). After 15 min of incubation at 65 °C, the eluate was collected by centrifugation at 10,000 *g* for 1 min. A rinse of the beads was performed by adding 50 μl of SDS sample buffer at 95 °C and centrifuging for 1 min at 10,000 *g*. Eluates were pooled into the ‘bound fraction' (elu). Corresponding amounts of bound (elu) and unbound (flow through, fth) fractions were analysed to allow direct comparability. Aliquots (10% of the total sample volume) of unbound or bound fraction and the corresponding inputs were analysed by SDS/PAGE on 4–12% NuPAGE Bis-Tris gels (Invitrogen) in NuPAGE MES SDS running buffer and the gel was stained with Bio-Safe Coomassie stain.

F-actin cosedimentation assays (Fig. 2c) were performed using α-actin (MA-99) according to the instructions of Cytokeleton, Inc supplied with the ‘Actin binding Protein Biochem Kit Muscle actin' with the here specified modifications. Twenty microlitres of a 48 μM actin (MA-99) solution in G'-buffer (5 mM Tris-HCl pH 8.0, 0.2 mM CaCl_2_, 0.5 mM DTT, 0.2 mM ATP, 5% glycerol) were thawed on ice, then added to 50 μl 50 mM bis-tris propane pH 9.5 and allowed to sit on ice for 40 min before polymerization was induced according to the protocol. These specific buffer conditions were chosen to allow maximum solubility of ExoY and were shown in our experiment to be compatible with polymerization of actin. Thirty microlitres of polymerized F-actin stock solution were combined with 20 μl of a solution containing 12 μg ExoY-FH in 50 mM bis-tris propane pH 9.5, 270 mM NaCl, 2 mM DTT and 1 × polymerization buffer from which non-soluble aggregates had been removed previously by centrifugation at 54,000 r.p.m. in a TL55 rotor (Beckmann) at 18 °C for 1 h. This mixture as well as controls containing only actin or only ExoY and the corresponding buffers present in the experiment were incubated at room temperature for 30 min and centrifuged at 54,000 r.p.m. for 90 min at 18 °C. Aliquots of supernatant and resuspended pellet fraction corresponding to 15% of the total samples were analysed by SDS–PAGE.

To measure the equilibrium dissociation constant (*K*_d_) of the ExoY:F–actin complex by cosedimentation assays we used MBP-ExoY/ExoY^K81M^-ST and muscle α-actin (MA-L) (Fig. 4c, Supplementary Fig. 6b). MBP-ExoY/ExoY^K81M^-ST should provide a reliable estimate of ExoY affinity for F-actin because these constructs perform similarily as ExoY/ExoY^K81M^-ST in depolymerization assays. MBP-ExoY/ExoY^K81M^-ST allowed separating and quantifying unambiguously by densitometry the fraction of the bound toxin at 88.9 kDa from actin at 42 kDa on SDS–PAGE gels, while ExoY-ST (M.W. of 43 kDa) was migrating too close to actin (M.W. of 42 kDa). No bundling activity was observed for ExoY in low-speed pelleting assays with F-actin. Three micromolar of F-actin-ADP-BeF_3_^−^ at steady state in (5 mM Tris-HCl pH 7.8, 0.1 M KCl, 2 mM MgCl_2_, 1 mM DTT, 5 mM ADP, 1.2 mM NaF, 0.6 mM BeCl_2_) (Fig. 4c) or of F-actin polymerized overnight to steady state in (5 mM Tris-HCl pH 7.8, 0.1 M KCl, 2 mM MgCl_2_, 1 mM DTT, 5 mM ATP, 2 mM GTP) with or without 6 μM phalloidin (Supplementary Fig. 6b) was mixed for 1 h with increasing amounts of MBP-ExoY/ExoY^K81M^-ST (0 to 17.9 μM). The unpolymerized (S, supernatant) and polymerized (P, pellet) fractions were separated by an ultracentrifugation for 30 min at 200,000 *g*, resolved by 10/15% SDS–PAGE and detected by coomassie blue staining. The ExoY-bound fraction was quantified by densitometry using the ImageJ software and this ExoY-bound concentration normalized by the F-actin concentration was plotted versus ExoY concentration. The following equation was used to fit the data, in which [F0] is the initial concentration of F-actin, [E0] the total concentration of ExoY in each measurement, and *K*_d_ the equilibrium dissociation constant. The fraction R of ExoY bound to F-actin is as follows:


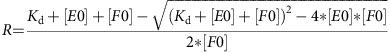


### Quantification of cAMP or cGMP synthesis *in vitro*

cAMP and cGMP synthesis were measured in 50 μl reactions containing 50 mM Tris-HCl pH 8.0, 7.5 mM MgCl_2_, 0.5 mg ml^−1^ BSA, 200 mM NaCl, 1 mM DTT, 2 mM ATP or GTP spiked with 0.1 μCi of [α-^33^P] ATP or [α-^33^P] GTP, respectively, ExoY and indicated amounts of HeLa/*S. cerevisiae* cell extracts or purified actin (collectively termed activator). Reactions for Figs 1a and 3a contained in addition 0.02% triton X-100, 0.1 mM CaCl_2_ and were lacking NaCl. Reactions were performed at 30 °C and were started by adding nucleotide substrate after a 5–10 min preincubation of ExoY plus activator. Under the conditions used, reactions were time linear for at least 20 min. Reactions were stopped by the addition of 450 μl stop solution (20 mM HEPES pH 7.5, 20 mM EDTA, 0.5 mM cAMP) and the mixtures were filtered on Al_2_O_3_ columns, which included three washes with 1 ml of 20 mM HEPES pH 7.5 each to separate nucleotide substrates that were retained in the columns from cyclic nucleotides present in the filtrates. Filtrates were collected in 20 ml scintillation vials. Sixteen millilitres scintillation liquid (HiSafe3, Perkin Elmer) were added before measuring ^33^P in a TriCarb scintillation counter (Perkin Elmer). All reactions were performed in duplicates. Differences between c.p.m. values of most duplicates were around or <10%. S.d.'s between duplicates are indicated by error bars.

Muscle actin 99% pure (designated MA-99) from rabbit skeletal muscle (Reference AKL99), or 99% pure non-muscle actin from human platelets (Reference APHL99, designated A-99) was obtained from Cytoskeleton, Inc. Alternatively, we used actin from rabbit skeletal muscle prepared in one of our laboratories (designated MA-L) according to the procedure described^48^. For activity assays, all actin solutions were diluted in G-buffer supplemented with BSA at 0.1 mg ml^−1^.

Preliminary experiments to optimize reaction conditions showed that ExoY-FH was most active at pH values between 8 and 9 and in Tris-HCl as compared with HEPES or Na-phosphate and shows a broad optimal NaCl concentration (between 100 and 300 mM NaCl).

Extracts from HeLa cells for activation of ExoY were prepared as follows: Cells grown in Dulbecco's modified Eagle's medium (DMEM)+10% fetal bovine serum were harvested after reaching 75% of confluence. One wash with PBS was followed by incubation in 10 ml PBS containing 0.01M EDTA for 5 min at 37 °C before detaching the cells by gentle tapping of the flasks. Cells were collected by centrifugation and washed three times in PBS. The cell pellet was resuspended in 2 ml of lysis buffer (50 ml Tris-HCl pH 7.5, 300 mM NaCl, 0.5% NP50, complete EDTA-free protease inhibitor cocktail (Roche)) per ml of cell pellet volume, after which the cells were snap frozen in liquid nitrogen and stored at −80 °C or processed immediately. Frozen cells were allowed to thaw on ice, rotated at 4 °C for 20 min and centrifuged for 1 h at 18,000 r.p.m. in a SS34 rotor (Sorval). The supernatant was centrifuged at 100,000 r.p.m. at 4 °C for 1 h in a TLA-110 rotor (Beckmann). The resulting supernatant was filtered through a 0.45 μM durapore polyvinylidene difluoride filter unit (Millipore) and dialysed against Tris-HCl/Triton/Glycerol buffer (TTG-buffer) containing 25 mM Tris-HCl pH 8.0, 0.1% Triton X-100, 10% glycerol, 1 mM DTT, 0.4 mM phenylmethylsulphonyl fluoride). Insoluble material was removed by centrifugation at 18,000 r.p.m. in a SS34 rotor at 4 °C for 20 min. HeLa cell extract prepared according to this protocol contained approximately 10 mg ml^−1^ of protein and was stored in aliquots at −20 °C.

Extracts from *S. cerevisiae* were prepared from 100 ml cultures grown in yeast extract peptone dextrose to an OD600 between 0.5 and 2, at which cells were harvested by centrifugation, washed once with water, and resuspended in 300 μl yeast lysis buffer (50 mM Tris-HCl pH 7.4, 50 mM KCl, 1 mM DTT, complete EDTA-free protease inhibitor cocktail (Roche)). Cells were subsequently vortexed for a total of 5 min (five times 1 min to prevent overheating) at 4 °C. Debris were removed by centrifugation at 16,000 r.p.m. for 15 min at 4 °C. The resulting extract contained about 4 mg ml^−1^ protein and was stored at −20 °C after the addition of glycerol to a final concentration of 10%.

Cycles of freezing and thawing did not seem to affect the activity of the cofactor necessary for ExoY activation in extracts from HeLa cells or *S. cerevisiae*.

Mg-ATP-actin was prepared from MA-L as follows: 90 μl of MA-L at 22.22 μM were added to 10 μl of 10 × concentrated buffer resulting in a final concentration of 40 μM MgCl_2_ and 0.4 mM EGTA and incubated for 10 min at room temperature and put on ice.

A 34 μM solution of Mg-ADP-actin was prepared as follows: Ca-ATP-actin (MA-L) was converted to Mg-ATP-actin by adding 100 × concentrated buffer resulting in a final concentration of 40 μM MgCl_2_ and 0.2 mM EGTA and incubating for 10 min at room temperature. In all, 15 U ml^−1^ of Hexokinase (Roche) was added together with glucose to a concentration of 5 mM followed by 30 min incubation on ice. The hexokinase inhibitor P1,P5-Di(adenosine*-*5′)pentaphosphate (Ap5A) was then added to 10 μM and the mixture was incubated for 5 min on ice. ADP and TCEP (tris(2-carboxyethyl) phosphine) was added to 0.2 and 2 mM, respectively. Mg-ADP-actin was diluted in G-ADP-buffer (5 mM Tris-HCl pH 7.8, 0.2 mM ADP, 2 mM TCEP, 30 μM MgCl_2_). Fifteen microlitres of the diluted actin solutions (Mg-ATP-actin or Mg-ADP-actin) were combined with 30 μl of a mixture containing 1 ng ExoY in reaction buffer to achieve final reaction buffer conditions of 50 mM Tris-HCl pH 8.0, 200 mM NaCl, 7.5 mM MgCl_2_, 2 mM DTT and 0.5 mg ml^−1^ BSA.

Studies on the effect of profilin or latrunculin were performed with actin (MA-L) polymerized to steady state. For studies on the effect of profilin: (1) control reactions: 5 μl of 5 × concentrated buffer resulting in a final concentration of 45 μM MgCl_2_ and 0.4 mM EGTA were added to 20 μl actin at 16.7 μM (diluted in G-buffer) and incubated for 10 min at room temperature. Twenty-five microlitres fresh 2 × polymerization buffer (F2: 300 mM KCl, 40 mM MgCl_2_, 10 mM ATP and 10 mM DTT) was added and samples were allowed to sit at room temperature for 2 h to allow polymerization to proceed to steady state. Varying amounts of the so-prepared F-actin were combined with F1-buffer (F1: 150 mM KCl, 20 mM MgCl_2_, 5 mM ATP and 5 mM DTT) to a total volume of 15 μl and added to 30 μl of a mixture containing 1 ng ExoY in buffer to achieve final reaction buffer conditions of 50 mM Tris-HCl pH 8.0, 200 mM NaCl, 7.5 mM MgCl_2_, 2 mM DTT and 0.5 mg ml^−1^ BSA. After 30 min preincubation at 30 °C, reactions were started by the addition of GTP (2 mM, spiked with [α-^33^P] GTP) and allowed to proceed for 10 min. (2) Reactions containing profilin: profilin was dialysed against G-buffer to remove the KCl present in the storage buffer before adding 5 μl at 142.7 μM directly to undiluted actin (6 μl MA at 55.88 μM in G-buffer), incubated at room temperature for 10 min and diluted by adding 14 μl G-buffer. Actin was not converted into Mg-ATP-actin. Twenty-five microlitres fresh F2 buffer was added and 11.2, 7.5 or 5.6 μl of this mixture were combined with G-buffer to a total volume of 15 μl and used immediately in activity assays. At final actin concentrations of 1.5, 1 and 0.75 μM, profilin was present at 3, 2 and 1.5 μM, respectively.

Latrunculin A was purchased from tebu-bio (produced by Focus Biomolecules). Studies with latrunculin were done similarly to those on profilin except that higher concentrations of actin (between 5.25 and 0.065 μM final MA-L) were used and reaction buffer did not contain BSA. Reactions containing latrunculin were converted into Mg-ATP-actin after combining actin and latrunculin (in twofold access over actin) by 10 min incubation at room temperature. Samples containing latrunculin were incubated 2 h at room temperature as the corresponding controls. The mixture of latrunculin and actin was diluted to different concentrations in G-buffer containing latrunculin to ensure equal concentrations of latrunculin in all reactions. Control reactions lacking latrunculin contained dimethylsulphoxide at concentrations equivalent to that introduced with latrunculin.

Inhibition of actin assembly by latrunculin was verified by cosedimentation assays (Supplementary Fig. 3).

### Pyrene-actin polymerization and depolymerization assays

Actin polymerization or depolymerization were monitored at 25 °C by the increase or decrease in fluorescence, respectively, of 3–10% (polymerization) or 50% (depolymerization) pyrenyl-labelled actin (*λ*exc=340 nm, *λ*em=407 nm). Actin-Ca-ATP in G-buffer was converted just before the experiments into G-actin-Mg-ATP by adding 1/100 (vol./vol.) of (2 mM MgCl_2_, 20 mM EGTA). Polymerization assays were performed in a final F0-buffer containing (0.1 M KCl, 2 mM MgCl_2,_ 30 mM Tris-HCl pH 7.8, 1 mM TCEP, 1 mM DTT), 5 mM ATP or 2 mM ADP and 4 mM GTP, unless indicated otherwise in figure legends. Polymerization with Arp2/3 (Fig. 4d) and depolymerization were performed in a final F1-buffer containing (0.1 M KCl, 8 mM MgCl_2,_ 50 mM Tris-HCl pH 7.8, 9 mM TCEP, 1 mM DTT, 0.3 mM (NH_4_)_2_SO_4_), 10 to 15 mM ATP or ADP, and 3 to 5 mM GTP, unless indicated otherwise in figure legends. Fluorescence measurements were carried out in a Safas Xenius model FLX (Safas, Monaco) spectrophotometer, using a multiple sampler device. Dilution-induced depolymerization assays were performed by quickly diluting 4 to 68 μl of 9 to 13 μM 50% pyrenyl-labelled F-actin at steady state into a final volume of 160 μl containing F1 buffer, ATP and the proteins of interest. Different fluorescence intensity levels were used in the two depolymerization assays of Fig. 4b.

### Random mutagenesis

Random mutagenesis was performed using the GeneMorph II kit (Agilent) according to the instructions of the manufacturer. Error prone PCR was performed using primers 1060 and M13R on template p1387. ClaI/SalI-digested fragments were cloned into plasmid p1182 digested with the same enzymes and transformed into *E. coli*. Transformants (≈4,000) were pooled for plasmid DNA extraction, which was subsequently transformed into *S. cerevisiae* SC483 to substitute wild-type actin with mutant alleles. This haploid *S. cerevisiae* strain lacks the chromosomal *act-1* gene and contains plasmid p1177, which harbours the wild-type *act1* gene and a Ura3 marker^35^. Initial transformants were harvested and pooled after 2 days incubation at 30 °C and re-plated on medium containing 5-fluoroorotic acid to select against the plasmid coding for wt actin. Colonies were pooled and kept as frozen stock at -80 °C. An aliquot of this mutant pool stock solution was transformed in batch with plasmid p1654 coding for Myc-ExoY-NanoLuc and plated on SG-agar supplemented with 0.05% glucose. Colonies were picked after 3–4 days and streaked on SG-agar. Forty of the initial 50 colonies survived under these conditions and were analysed for expression of ExoY by anti-Myc western blots. Plasmid DNA from the 21 clones that did not show impairment of ExoY expression was transformed into *E. coli* selecting for the kmR marker of the plasmids carrying the actin mutant allele. Sequences of the *act-1* mutant alleles were then analysed.

Isolated plasmids (p1688 and p1689) carrying either one of the two distinct mutant alleles that were found (D25Y/D222G and D25N, respectively) as well as p1387 (wild-type actin) were transformed into SC483 to ensure a clean strain background. The strains obtained after elimination of the wild-type *act1* gene (SC690, SC691, and SC489) were used in stress tests to study potential effects of the actin mutant alleles on growth under different conditions and effects on cytotoxicity of ExoY expression.

### Light microscopy and quantifications of F-actin content

HeLa cells were transiently transfected with AcGFP or ExoY^K81M^-AcGFP constructs. Transfection with ExoY^wt^ construct resulted in cell death, which precluded the use of ExoY^wt^. Thirty-six hours later, cells were trypsinized and replated on fibronectin coated coverslips for 2 h. After fixation and permeabilization, cells were stained using Acti-stain 670 fluorescent phalloidin (Cytoskeleton, Inc.). Confocal images were acquired by a Nipkow Spinning Disk Confocal system (Yokogawa CSU-X1-A1) mounted on an inverted microscope (Nikon Eclipse Ti-E) using a × 100 Apochromat TIRF oil-immersion objective (NA: 1.49). Blue (491 nm Cobolt Calypso) and red (642 nm Toptica iBeam Smart 640-S) lasers were used for excitation of AcGFP and 670 nm fluorescent phalloidin, respectively. Images were recorded with an ORCA-Flash4.0 LT CMOS camera (Hamamatsu) and stacks were acquired with a step of 300 nm using MetaMorph 7.7 (Molecular Devices) under identical settings. The cross correlation analysis on the co-localization data was performed using PCC measured from AcGFP and 670 nm fluorescence inside the successive sections of the individual cells. The PCC is a quantitative measurement that estimates the degree of overlap between fluorescence signals obtained in two channels^58^. The quantification of the fluorescence intensity of stress fibres was obtained by analysing the bottom three slices after subtraction of background from the nearby cytoplasm. Total amount of F-actin was assessed by the intensity of phalloidin staining in the whole cell. Image analyses (region of interest selection as contours of the whole individual cells, intensity measurements, co-localization analyses) were performed using Icy software (Institut Pasteur, France Bio Imaging)^59^. Statistical analysis was performed using Student's *t-*test as significance test, with *P*≤0.001.

For TIRF microscopy, HeLa cells were cultured in DMEM, 5% CO_2_. Cells were transiently transfected with vinculin-mCherry^60^ and either GFP-C1 (Clontech, Palo-Alto, CA, USA) or ExoY^K81M^-AcGFP using Lipofectamine (Thermo Fisher, Carlsbad, CA, USA) as per the manufacturer's instructions. Cells were plated onto plasma cleaned coverslips 24 h before imaging. Images were acquired using a custom built TIRF microscope.

### Data availability

The authors declare that the data supporting the findings of this study are available within the paper and its Supplementary Information files.

## Additional information

**How to cite this article:** Belyy, A. *et al*. Actin activates *Pseudomonas aeruginosa* ExoY nucleotidyl cyclase toxin and ExoY-like effector domains from MARTX toxins. *Nat. Commun.* **7,** 13582 doi: 10.1038/ncomms13582 (2016).

**Publisher's note:** Springer Nature remains neutral with regard to jurisdictional claims in published maps and institutional affiliations.

## Supplementary Material

Supplementary Information Supplementary Figures 1-10, Supplementary Tables 1-2 and Supplementary References

Supplementary Data 1 Mass spectrometry based quantitation (LFQ scores) of proteins copurifying from yeast with ExoY-TAP.

## Figures and Tables

**Table 1:** Activation of ExoY or VnExoY-L by *S. cerevisiae* extracts expressing wild-type or mutant actin.

	**No extract**	***S. cerevisiae*** **extracts**		
		**SC489 Wild type**	**SC690 D25Y/D222G**	**SC691 D25N**
ExoY-catalysed synthesis of cGMP (nmol min^−1^ mg^−1^)	<5	2,950±330	<5	<5
VnExoY-L-catalysed synthesis of cAMP (nmol min^−1^ mg^−1^)	<5	2,800±210	<5	19±8

Reactions containing 0.5 μg of ExoY-FH or VnExoY-L-FH and 50 μg of *S. cerevisiae* cell extracts were started by the addition of 2 mM GTP or ATP substrate and incubated for 30 min at 30 °C.
