# Supplementary material for: Actin activates Pseudomonas aeruginosa ExoY nucleotidyl cyclase toxin and ExoY-like effector domains from MARTX toxins
Source: Nat Commun. 2016 Dec 5;7:13582. doi: 10.1038/ncomms13582 (PMC5150216; doi:10.1038/ncomms13582)
Supplement: Supplementary Information — Supplementary Figures 1-10, Supplementary Tables 1-2 and Supplementary References [file ncomms13582-s1.pdf]

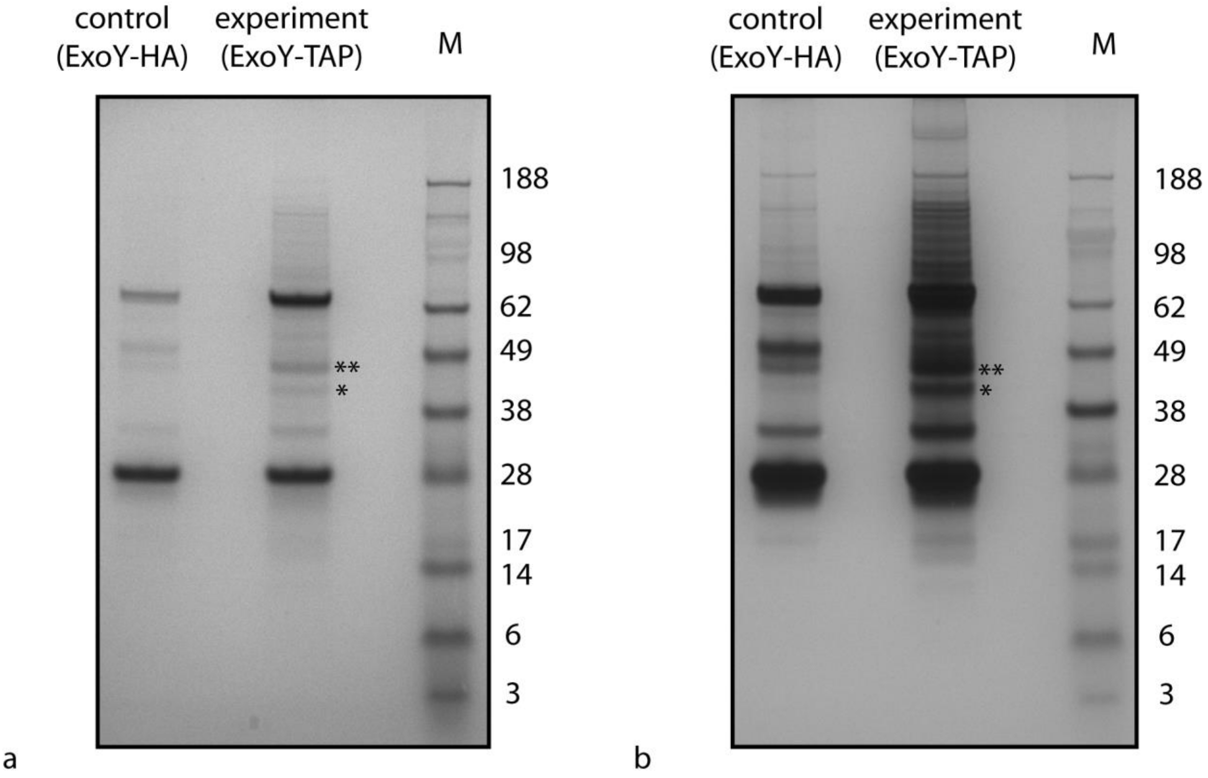

Supplementary Figure 1. Affinity purification of ExoY^{K81M}-TAP expressed in *S. cerevisiae* with potential interacting proteins. ExoY^{K81M}-HA expressing cells were used as control purification. Shown is the separation of samples by SDS-PAGE on 4-12 % NuPAGE® Bis-Tris gels (Invitrogen) in NuPAGE® MES-SDS running buffer. The gel was stained with **(a)** Bio-Safe Coomassie G-250 (BIO-RAD) followed by **(b)** silver staining (Pierce). Bands with a molecular weight of around 42 kD (*) and 49 kD (**) correspond likely to actin and ExoY (after digestion with TEV protease and still containing the calmodulin binding peptide), respectively.

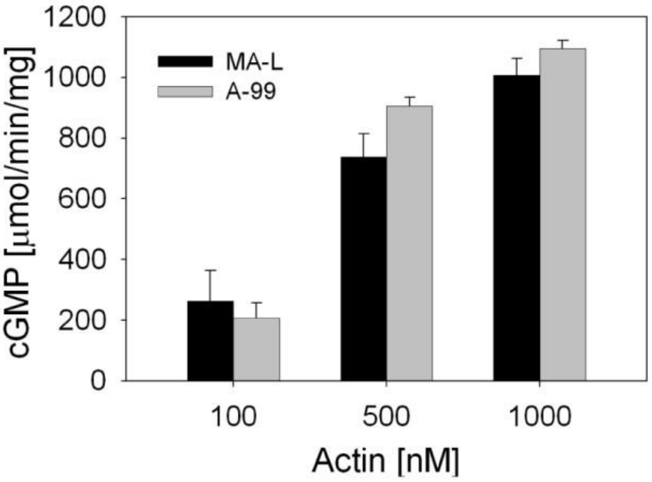

Supplementary Figure 2. Activation of ExoY-catalyzed cGMP synthesis by different isoforms of actin. We compared ExoY activity in the presence of different actin isoforms. Actin from human platelets (A-99), a mixture of 85% β- and 15% γ-actin obtained from Cytoskeleton, Inc., and muscle α-actin purified in our laboratory from rabbit skeletal muscle (designated MA-L). ExoY-FH at 0.5 nM (1 ng) was activated by commercially available non-muscle actin A-99, or muscle actin MA-L at 100, 500 or 1000 nM. The cGMP

synthesis activity of ExoY was strongly activated in a concentration dependent manner by both actin isoforms tested indicating that they were effective activators of ExoY. Subsequent experiments were performed using α-actin from rabbit skeletal muscles purified in our laboratory and fully functional in actin polymerization assays (>95% pure, designated MA-L). Error bars correspond to s.d. of 2 experimental replicates.

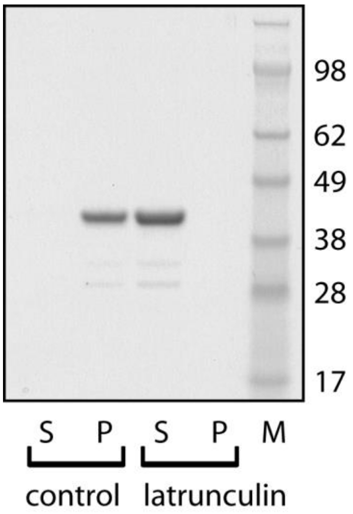

Supplementary Figure 3. Inhibition of actin polymerization by latrunculinA. Using high-speed (100 000*g) sedimentation assays, we verified that latrunculin indeed inhibited actin polymerization. Stock solutions used for activity measurements in Fig. 3c contained 17.5 μM Mg-ATP-actin that was prevented from polymerizing by the presence of 38.6 μM latrunculin A or controls (containing DMSO) polymerized to steady state by the addition of salt. Aliquots of supernatant (S) or pellet (P) fractions after ultracentrifugation were separated by SDS-PAGE.

a **c**

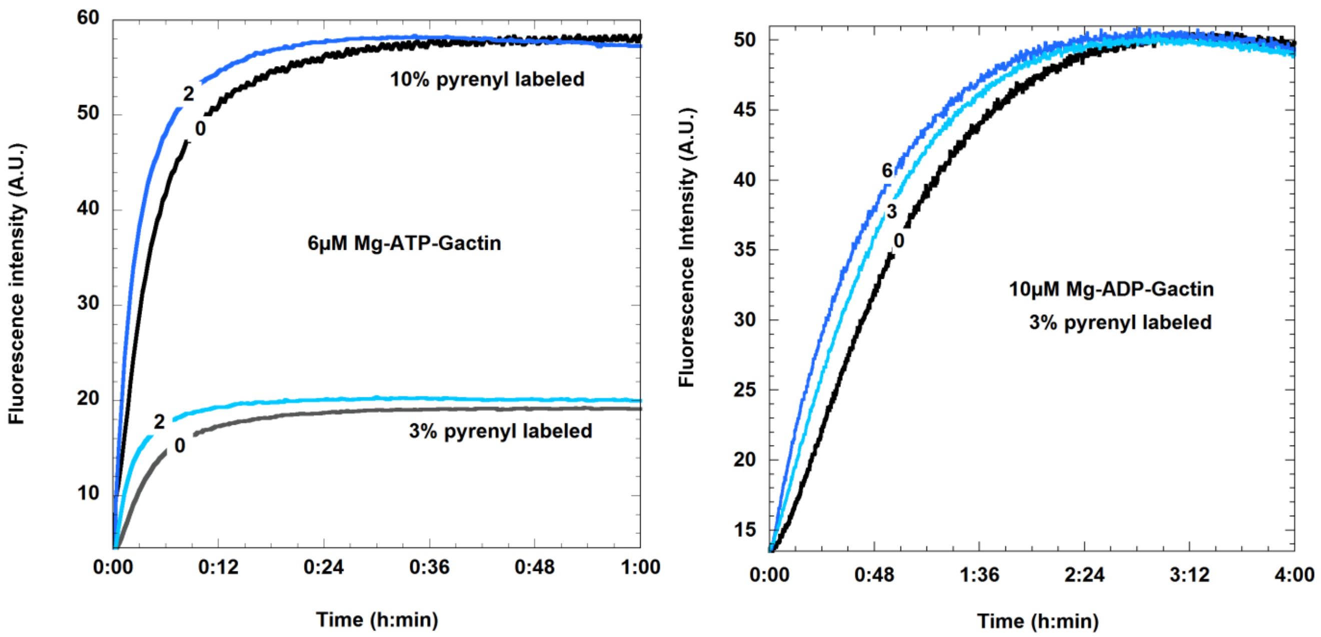

b

[ExoY] (µM)	t1/2 (sec) = time at which half of the maximum fluorescence intensity is reached	rates of bulk actin polymerization at 50% saturation (µM/sec)
**0**	5253	23.23
**0.25**	4679	22.79
**0.5**	3923	19.35
**1**	3428	15.62
**2**	2648	11.48
**3**	2547	10.00
**4**	2329	9.38
**8**	2091	8.16

Supplementary Figure 4. ExoY interaction with actin does not significantly affect the fluorescence of F-actin at 3 or 10% pyrene labeling (a) and slightly accelerates the rate of G-actin-ATP/ADP-Mg self-assembly (b). (a) 6 µM G-actin-Mg-ATP (10 or 3% pyrenyl labeled) was polymerized in the absence (black curves) or presence of 2 µMExoY^{K81M} (blue curves). Similar final pyrene fluorescence intensity was obtained at steady state in the absence or presence of ExoY. **(b)** The table reports times at which half of the maximum fluorescence intensity is reached and the rates of bulk actin polymerization at 50% saturation in the absence or presence of 0.25 to 8 µM ExoY^{K81M} as in Fig. 4a (polymerization of 4 µM G-actin-Mg-ATP, 3% pyrenyl labeled), showing the dose-dependent acceleration of G-actin-ATP polymerization induced by ExoY^{K81M}. **(c)** 10 µM G-actin-Mg-ADP (3% pyrenyl labeled) was polymerized in the absence (black curves) or presence of 3 and 6 µM ExoY (blue curves). Similar final pyrene fluorescence intensity was also obtained at steady state in the absence or presence of ExoY.

a b

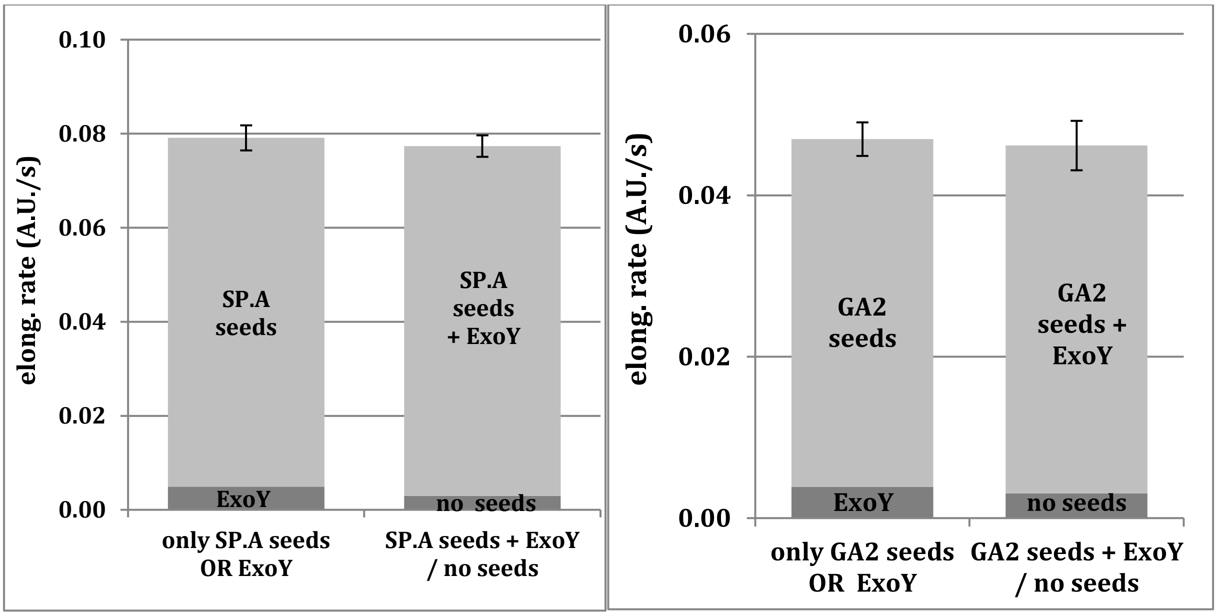

Supplementary Figure 5. ExoY does not accelerate the barbed- (a) or pointed-end (b) elongation rates of actin self-assembly, nor severs actin filaments to mediate acceleration of actin self-assembly at high concentrations. We measured the initial rates of 4 µM G-actin-Mg-ATP (10% pyrenyl labeled) self-assembly with 0 or 0.4 µM of ExoY in the absence and presence of pre-formed actin seeds with free barbed- or pointed-ends using spectrin-actin seeds (designated SP.A, 0.19 nM of SP.A seeds, panel (a) or Gelsolin-actin seeds capped at their barbed-ends by gelsolin (designated GA2, 4.7 nM of GA2 seeds, panel (b), respectively. Mixing ExoY and actin seeds does not accelerate the barbed- (a) or pointed-end (b) elongation rates of actin self-assembly, nor severs actin filaments. If ExoY severed or accelerated barbed- or pointed-end elongation, the polymerization rates in the presence of seeds would be higher. Dilution-induced depolymerization kinetics further confirmed that ExoY does not accelerate spontaneous filament disassembly as expected with severing, but instead inhibits spontaneous filament disassembly (Fig. 4b). Error bars represent standard deviations of four independent measurements.

a

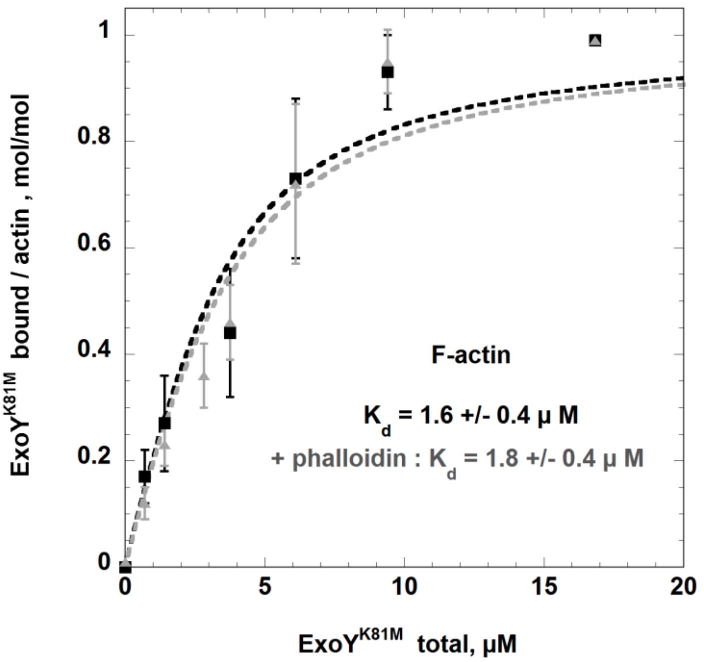
ExoY^{K81M} bound / actin , mol/mol
ExoY^{K81M} total, μM
F-actin
K_d = 1.6 +/- 0.4 μ M
+ phalloidin : K_d = 1.8 +/- 0.4 μ M
0
0.2
0.4
0.6
0.8
1
0
5
10
15
20

b

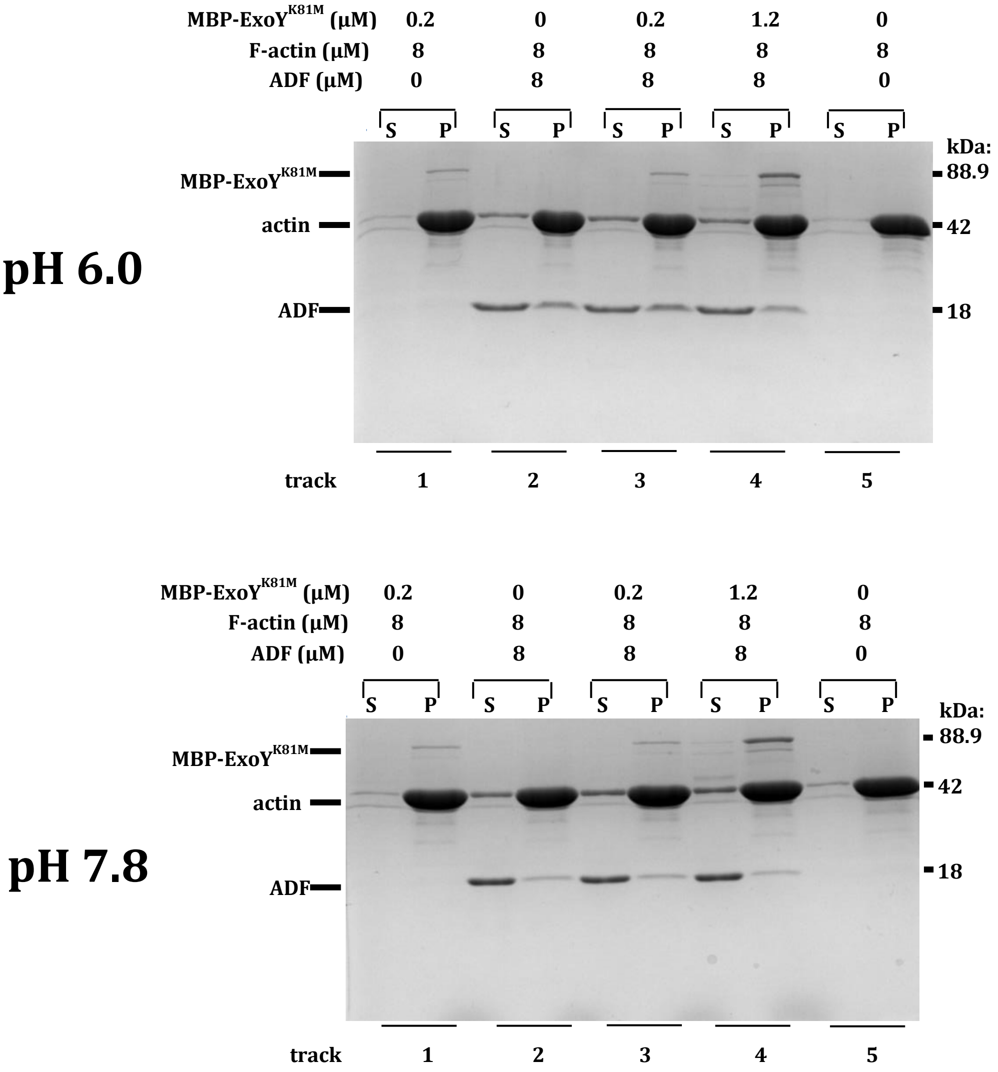
MBP-ExoY^{K81M} (μM) 0.2 0 0.2 1.2 0
F-actin (μM) 8 8 8 8 8
ADF (μM) 0 8 8 8 0
S P S P S P S P S P
kDa:
MBP-ExoY^{K81M}
actin
ADF
88.9
42
18
pH 6.0
track 1 2 3 4 5
MBP-ExoY^{K81M} (μM) 0.2 0 0.2 1.2 0
F-actin (μM) 8 8 8 8 8
ADF (μM) 0 8 8 8 0
S P S P S P S P S P
kDa:
MBP-ExoY^{K81M}
actin
ADF
88.9
42
18
pH 7.8
track 1 2 3 4 5

Supplementary Figure 6. **MBP-ExoY^{K81M} binds with a similar affinity to native and phalloidin-stabilized F-actin, and its binding to F-actin does not significantly inhibit the binding of ADF at the low MBP-ExoY^{K81M}: ADF ratios that antagonized ADF-mediated F-actin disassembly in Fig. 4e.**

(a) Binding affinity of MBP-ExoY^{K81M} for native or phalloidin-stabilized F-actin measured in high-speed cosedimentation assays as in Fig. 4c but with F-actin at steady state polymerized from G-actin-ATP with 5 mM ATP in absence or presence of phalloidin (in a 2-fold molar excess over actin). Error bars are standard errors (n≥3). The Kd of MBP-ExoY^{K81M} for native or phalloidin-stabilized F-actin is similar to its Kd for F-actin-ADP-BeF_3^- (Fig. 4c).

(b) Representative Coomassie blue stained SDS-PAGE (15%) gel images from the supernatant (S) and pellet (P) fractions of actin filaments in F-buffer at pH 6.0 (upper panel) or 7.8 (lower panel) pre-incubated with MBP-ExoY^{K81M} (30 min), prior to ADF incubation (1h), and ultracentrifugation (200,000 g, 30 min).

The cosedimentation assays were performed in conditions similar to Fig. 4e concerning the incubation time with ADF (1 h) and the low ExoY^{K81M}:ADF ratios that were sufficient to antagonize ADF activity in the depolymerization assays of Fig. 4e (ratios of 1:40 or 1:6.7). In contrast to experiment shown in Fig. 4e, we used non-labeled F-actin polymerized to steady state in a buffer containing ATP (5 mM) to follow ADF binding. ADF binds preferentially to “aged” ADP–actin filaments to stimulate filament severing or disassembly. The cosedimentation assays were additionally performed both at pH 7.8 (100 mM Tris-HCl) and 6.0 (100mM MES buffer). Below pH 7.3 ADF binds to F-actin but its depolymerizing activity is impaired[1],which allows to uncouple artificially binding and depolymerization[1],[2]. The amounts of unbound (S) ADF and bound to filaments (P) were measured by densitometry. At both pH values the addition of ADF to F-actin increased the proportion of actin in the supernatant fractions (track 2, S) compared to F-actin alone (track 5) or when pre-incubated with MBP-ExoY^{K81M} (track 1), and more significantly at pH 7.8 than at pH 6.0. At pH 6.0, the proportions of ADF that cosedimented with filaments (track 2, P) were higher than at pH 7.8 as expected[1]. At both pH values there was no significant loss of ADF binding to F-actin with a low MBP-ExoY^{K81M}:ADF ratio of 1:40 (ratio sufficient to antagonize ADF-mediated F-actin disassembly in Fig. 4e, corresponding here to 0.2 µM MBP-ExoY^{K81M} and 8 µM ADF, track 3 compared to track 2), and a loss of only 16% at a 1:6.7 ratio (maximal ratio used in Fig. 4e, corresponding here to 1.2 µM MBP-ExoY^{K81M} and 8 µM ADF, track 4 compared to track 2).

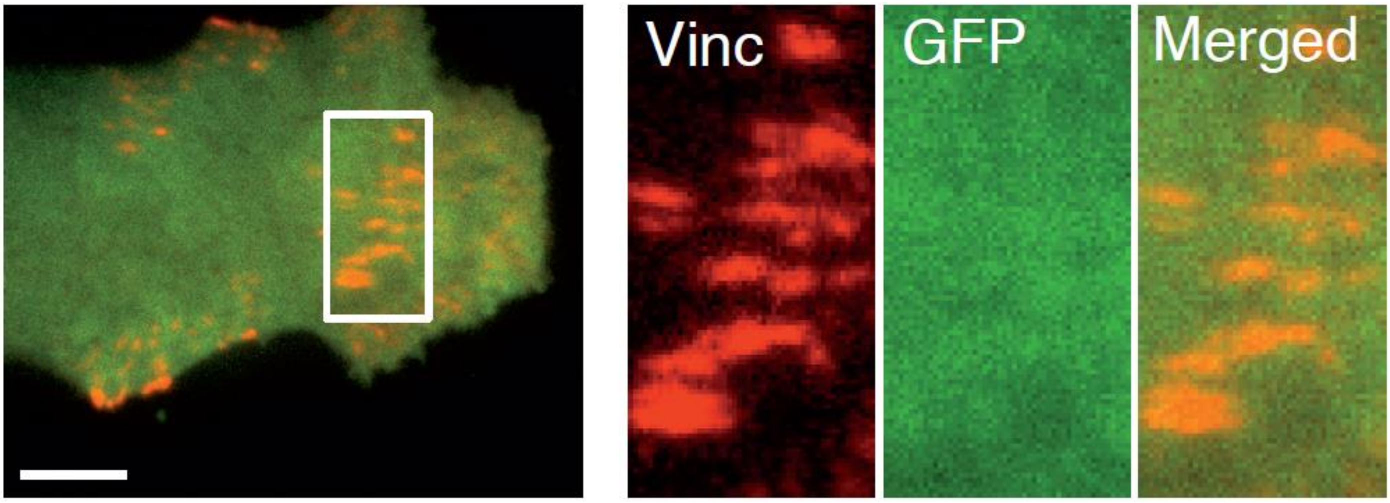

Supplementary Figure 7. TIRF picture of a control HeLa cell expressing GFP alone and mCherry-vinculin. GFP alone (green) was uniform and did not colocalize in the extension of vinculin (red) labelled focal adhesions. (scale bars, 5µm).

```
ExoY Pseu.aerug.11 ------------------------------------------------------------
ExoY_Pseu.aerug.2    1 ------------------------------------------------------------
ExoY-L.Prov.stuar    1 ------------------------------------------------------------
ExoY-L.Burk_pseud    1 ------------------------------------------------------------
ExoY-L.Prot.penne    1 ------------------------------------------------------------
ExoY-L.Vibr.chole    1 ---------------LDQLDLVHQTAGRDQIVASFELNKKVNAYIAEHPTSRRNQALTQL
ExoY-L.Vibr.nigri    1 --------------WRPKFGVWTPTELYNYGQALQEAQLDIATMKPRQRVTANELQLGDD
ExoY-L.Vibr.vulni    1 GEKLRAKLAIAGYNVSGEQTFYGHEASNRLMGQYADQIVSGLFNAEQAAVEVKDIRATED
ExoY-L.Prot.vulga    1 -------GVILSSYLPKNEFIFPSDLEINTTIYFSYENPNEDYNNNIIYNELLNKNRKVK
ExoY-L.Vibr.angui    1 ------MQGILDLPVFDATPIKKPGTSDVDAKVVDDTKEALADGKILHNQDVNDHDEITT
ExoY-L.Prot.mirab    1 IVLSSSNNDLILVVNKVKQLKYKTDIYIAHDHQITDENNFLIDRILKSFGHEVKKFSFSH
ExoY-L.Vibr.ordal    1 AVKETDQNLHIVAKYTEEFHAGETIRNLFDGKTIGMAINQNTSTINESVITSFDHDEITT
ExoY-L.Prov.rettg    1 ENCLLKIKVVTGSTEFIAYTDILDGQDLYTRIINNPETIAKIKKDVFISQDGYKIYSINK
CyaA_Bort.pertu.C    1 ------------------------------------------------------------
EF_Baci.anthr.Cyc    1 ------------------------------------------------------------

ExoY Pseu.aerug.11 --MRIDGHRQVVSNATAQPGPLLRPADMQARALQDLFDAQGVGVPVEHALRMQAVARQTN
ExoY_Pseu.aerug.2    1 --MRIDGHRQVVSNATAQPGPLLRPADMQARALQDLFDAQGVGVPVEHALRMQAVARQTN
ExoY-L.Prov.stuar    1 MIQTHKSYNKIGYEPLQAVNEPTEQKTTHSIAQLTECISQNTGLVSSHLLPLQQVAKEQD
ExoY-L.Burk_pseud    1 --------------------------------MLCARVQGETGVVPAHLVKLQRVAGEHN
ExoY-L.Prot.penne    1 -------------------------------------------MPIKDQKIFSQIAEEKG
ExoY-L.Vibr.chole   46 KEQITSALLNNETELKIESRPKNYQSRDLVLEPIVQPETIELGMPDIDQKILAEVAEREN
ExoY-L.Vibr.nigri   47 NAITNAVTSEQEATPNQDGSHKTYQSRDLVLEPIQHPKSIELGMPEVDQSVLAEVAEREN
ExoY-L.Vibr.vulni   61 LSVVKTVASDTELGTNTDAPHKNYQSRDLVLEPIVQPETIELGMPDSDQKILAEVAEREN
ExoY-L.Prot.vulga   54 KFSFTNREEYNSFSELNKIEQKKTLKSLGYNNKKNEINEIKIGMPIKDQKIFSQIAEEKG
ExoY-L.Vibr.angui   55 LLKNKPVLDDAETISNADSHHKNYQSRDLVLEPIVQPETIELGMPDIDQKILAEVAEREN
ExoY-L.Prot.mirab   61 REEYNNFYNCKYHQPDPVRKFSIYQSRNLTLAPIEQEPIPTIGMPKNDQKAFRQAADSEN
ExoY-L.Vibr.ordal   61 LLKNKPVLGDAETVSNADSRHKSYQSRDLVLEPIVQPETIELGMPDIDQKILAEVSEREN
ExoY-L.Prov.rettg   61 NFDIKTFENINKSHNNQNIARDYIRTNLHLKPLSSNEPKAITGIPENHQRAITQVANDYN
CyaA_Bort.pertu.C    1 -------------------------MQQSHQAGYANAADRESGIPAAVLDGIKAVAKEKN
EF_Baci.anthr.Cyc    1 ---------------------------DRIDVLKGEKALKASGLVPEHADAFKKIARELN
```

catalytic deficient mutant
of *P. aeruginosa*ExoY: K81M

motif I

```
ExoY Pseu.aerug.1   59 TVFGIRPVERIVTTLIEEG-FPTKGFSVKGKSSNWGPQAGFICVDQHLSKREDRDTAEIR
ExoY_Pseu.aerug.2   59 TVFGIRPVERIVTTLIEEG-FPTKGFSVKGKSSNWGPQAGFICVDQHLSKRENRDTAEIR
ExoY-L.Prov.stuar   61 SIIGVRPVDRFATDLIAAG-YPTKNFHIKGKSASWGAQAGLICVDQRFSKLENKSEDLID
ExoY-L.Burk_pseud   29 CLIGIRPVDRFATDLIESG-YPTKGFHIKGKSANWGPQAAFICVNQQFSKLEDKPD-RIE
ExoY-L.Prot.penne   18 IIIGVRPIDANSTSLISSGEYSSKNLAIKAKSSDWGPMAGFIPVNQSLAKASAQK--NLL
ExoY-L.Vibr.chole  106 VIIGVRPVDEKSKSLIDSKLYSSKGLFVKAKSSDWGPMSGFIPVDQVFAKASARR--DLD
ExoY-L.Vibr.nigri  107 VIIGVRPVDEKSKSLIASKMYSSKGLFVKAKSSDWGPMSGFIPVDQSFAKASARR--DLE
ExoY-L.Vibr.vulni  121 VIIGVRPVDEKSKSLIDSKLYSSKGLFVKAKSSDWGPMSGFIPVDQAFAKASARR--DLD
ExoY-L.Prot.vulga  114 IIIGVRPIDANSTSLISSGEYSSKNLAIKAKSSDWGPMAGFIPVDQSLAKASAQK--NLL
ExoY-L.Vibr.angui  115 VIIGVRPVDEKSKSLIDSKLYSSKGLFVKAKSSDWGPMSGFIPVDQSFAKASARR--DLD
ExoY-L.Prot.mirab  121 VIIGVRPIDARSTTLIQSREYSSKGLLIKGKSSDWGPMAGFIPVDQSLAKLSARN--NVA
ExoY-L.Vibr.ordal  121 VIIGVRPVDEKSKSLIDSKLYSSKGLFVKAKSSDWGPMSGFIPVDQSFAKASARR--DLD
ExoY-L.Prov.rettg  121 VIIGMRPIDYKSTSLIASGLYGSKGLTIKGKSSDWGPHSGFIPILQQFAKKSGRD--EQE
CyaA_Bort.pertu.C   36 ATLMFRLVNPHSTSLIAEG-VATKGLGVHAKSSDWGLQAGYIPVNPNLSKLFGRAPEVIA
EF_Baci.anthr.Cyc   34 TYILFRPVNKLATNLIKSG-VATKGLNVHGKSSDWGPVAGYIPFDQDLSKKHGQQL-AVE
```

```
ExoY_Pseu.aerug.1  118 KLNLAVAKGMD--GGAYTQTDLRISRQRLAELVRNFG-------LVADGVGPVRLLTA-Q
ExoY_Pseu.aerug.2  118 KLNLAVAKGMD--GGAYTQTDLRISQQRLAELVRNFG-------LVADGVGPVRLLTA-Q
ExoY-L.Prov.stuar  120 KYNGFIRRCIE--KGHARPIPLEITEERLHTLLQM-GAI-D-NLSLENSQGVKQFTAK-G
ExoY-L.Burk_pseud   87 KFNRQVQSCIA--EGHAKSVLLMLSQARLEKLLDE-GLIDKVQFGRNRVPIMFEAKAP-S
ExoY-L.Prot.penne   76 KYNKAVEEAIN--SGAASVSELYISAERVNELIEN-KVI----DYLYDKNSPIKFKVIHN
ExoY-L.Vibr.chole  164 KFNGYAEQSIE--SGNAVSADLYLNQVRIDELVSKYQSL-T-ALEFDAESGMYKTTATNG
ExoY-L.Vibr.nigri  165 KFNEYAEQSIL--SGNAVSANLYLNQVRIEELVSKYESL-T-PLELDVDSGMYKTTATNG
ExoY-L.Vibr.vulni  179 KFNGYAEQSIE--SGNAVSADLYLNQVRIDELVSKYQSL-T-ALEFDAESGMYKTTATNG
ExoY-L.Prot.vulga  172 KYNKAVEEAIN--SGAASVSELYISAERVNELVEN-KVI----DYLYDKNSPIKFEVIHN
ExoY-L.Vibr.angui  173 KFNGYAEQSIE--SGNAVSADLYLNQVRMDELVSKYQSL-T-ALEFDAESGMYKTTATNG
ExoY-L.Prot.mirab  179 KYNLANQEALD--KGHAISVPLTLSPERVRELQQY-NIL----NYSDLLTDIIQVTCRVD
ExoY-L.Vibr.ordal  179 KFNGYAEQSIE--SGNAVSADLYLNQVRIDELVSKYQSL-T-ALEFDAESGMYKTTATNG
ExoY-L.Prov.rettg  179 KYNGYITQSIE--QGHAKPVILEVTHERMNELLKY-GAV-TSLEDIGRGDHIRRTTAKVD
CyaA_Bort.pertu.C   95 RADNDVNSSLA--HGHT-AVDLTLSKERLDYLRQA-GLVTGMAD--------GVVASNHA
EF_Baci.anthr.Cyc   92 KGNLENKKSITEHEGEIGKIPLKLDHLRIEELKEN-GIILKGKKEIDNGKKYYLLESNN-
```

```
                                                                     motif II
ExoY_Pseu.aerug.1  168 GPSGKR-YEFEARQEPDGLYRISR--LG--RSEAVQVLASPA-C---GLAMTADYDLFLV
ExoY_Pseu.aerug.2  168 GPSGKR-YEFEARQEPDGLYRISR--LG--RSEAVQVLASPA-C---GLAMTADYDLFLV
ExoY-L.Prov.stuar  174 PSLKSY-QFEAKWEPQQQKYRI----YF--EKKYLQVLAPID-S---CLPITADYDLLLI
ExoY-L.Burk_pseud  143 GMVYEF-EAAHAYSAGEDMYKI----TH--GGTQIEVLAPPRDD---AKPLTADYDLFLI
ExoY-L.Prot.penne  129 NKTHEF-FLEPKIIENKQYYLVQYYNHS--GTEPVLVMSDPI-S---NKPMIADYDLFTV
ExoY-L.Vibr.chole  220 DQTATF-FLNKVTVDSKDLWQVHYMKDG--ELAPFKVIGDPV-S---KQPMTADYDLLTV
ExoY-L.Vibr.nigri  221 DQTIPF-FLNKVTVDDKELWQVHYLREG--ELAPFKVIGDPV-S---KQPMTADYDLLTV
ExoY-L.Vibr.vulni  235 DQTVTF-FLNKVTVDSKDLWQVHYIKDG--KLAPFKVIGDPV-S---KQPMTADYDLLTV
ExoY-L.Prot.vulga  225 NKTYEF-FLEPKIIENKQYYLVQYYNHS--GTEPVLVMADPI-S---NKPMIADYDLFTV
ExoY-L.Vibr.angui  229 DQTVTF-FLNKVTVESKDLWQVHYMKDG--ELAPFKVIGDPV-S---KQPMTADYDLLTV
ExoY-L.Prot.mirab  232 NKEYYF-YLSKVNQDGKVTYTVNSYDNG--KLIPVNVLADPI-S---KKPMIADYDLFTV
ExoY-L.Vibr.ordal  235 DQTVTF-FLNKVTVESKDLWQVHYMKDG--ELAPFKVIGDPV-S---KQPMTADYDLFTV
ExoY-L.Prov.rettg  235 NIPHSF-ILNNITRGETKLWQVFYQSGH--KTAPFLVVGDPK-T---GKAMTADYDLFSV
CyaA_Bort.pertu.C  143 GYEQFEFRVKETSDGRYAVQYRRKGGDDF---EAVKVIGNA--A---GIPLTADIDMFAI
EF_Baci.anthr.Cyc  150 --QVYEFRISDENNEVQYKTKEGKITVLGEKFNWRNIEVMAKNVEGVLKPLTADYDLFAL
```

```
ExoY_Pseu.aerug.1  219 APSIEAHGS----------------------------------GGLDARRNTAVRYTPLG
ExoY_Pseu.aerug.2  219 APSIEAHGN----------------------------------GGLDARRNTAVRYTPLG
ExoY-L.Prov.stuar  223 GPHMRDFGHQD----------------------MLPVPDVAHSVYRQRVEKYQRLPTDAN
ExoY-L.Burk_pseud  193 GPHITDLGPQD----------------------NLPVP-DVSHAVFKARLDKYKNGIPSA
ExoY-L.Prot.penne  182 IYPYSHLGTNT----------------------RVNKP--VSWEEWKQ----SVNYAELT
ExoY-L.Vibr.chole  273 MYSYADLGPQD----------------------KVKQP--LNWEQWKE----SVTYEDLT
ExoY-L.Vibr.nigri  274 MYTYGDLGPQD----------------------KVKQP--LTWEQWKE----SVTYEDLS
ExoY-L.Vibr.vulni  288 MYSYSDLGPQD----------------------KLKQP--LTWEQWKE----SVTYEELT
ExoY-L.Prot.vulga  278 IYPYSHLGTNT----------------------RVNMP--VSWEEWKQ----SVNYAELT
ExoY-L.Vibr.angui  282 MYSYADLGPQD----------------------KVKQP--LTWEQWKE----SVTYEDLT
ExoY-L.Prot.mirab  285 MYSYNDLSERS----------------------TVRKA--IPWEEWRS----SVNYDELS
ExoY-L.Vibr.ordal  288 MYSYAELGPQD----------------------KVKQP--LTWEQWKE----SVTYEDLT
ExoY-L.Prov.rettg  288 IYPVSELEHYV----------------------KVSEM--PSWQEWKA----SVNYDELT
CyaA_Bort.pertu.C  195 MPHLSNFRDSARSSVTSGDSVTDYLARTRRAASEATGG--LDRERIDL----LWKIARAG
EF_Baci.anthr.Cyc  208 APSLTEIKKQIQKEW---DKVVN------------------TPNSLEK----QKGVTNLL
```

```
ExoY_Pseu.aerug.1    245 A--KDPLSEDG-F--YGREDMARGNITPRTRQLVDALNDCLGRG--EHREMFHHSDDAGN
ExoY_Pseu.aerug.2    245 A--KDPLSEDG-F--YGREDMARGNITPRTRQLVDALNDCLGRG--EHREMFHHSDDAGN
ExoY-L.Prov.stuar    261 L--SQAYRDENHF--YQNEDSEIGNASPRVREMISLINRALV-G--EAEKVVHHSVDATS
ExoY-L.Burk_pseud    230 L--RSAYEQSGEF--YRKEDEEIGNASLRVRNMIPLINTALV-G--DGEAVVHHSTDSAS
ExoY-L.Prot.penne    214 DKQKILYNDKLLY--EKNEGHQLGFISQQIKELKDELNTALGRS--TGMEIVHHGADDAN
ExoY-L.Vibr.chole    305 PKYKELYNSEVLY--NKKDGASLGVVSDRLKALKDVINTSLGRT--DGLEMVHHGADDAN
ExoY-L.Vibr.nigri    306 PKYKARYDNQALY--EKQDGASLGMVSDRLKELKDVINTSLGRT--DGLEMVHHGADDAN
ExoY-L.Vibr.vulni    320 PKYKELYNSEVLY--NKKDGASLGVVSDRLKALKDVINTSLGRT--DGLEMVHHGADDAN
ExoY-L.Prot.vulga    310 DKQKILYNDRLLY--EKNEGHQLGFISQQIKELKDELNTALGRS--TGMEIVHHGADDAN
ExoY-L.Vibr.angui    314 PKYQELYDSEVLY--NKKDGASLGVVSDRLKTLKDVINTSLGRT--DGLEMVHHGADDAN
ExoY-L.Prot.mirab    317 PTYQEYYNNKDLY--DRYEGEQLGIISQHVKNIKNKLNRLLQRE--KGKEMIHHGADDAN
ExoY-L.Vibr.ordal    320 PKYKELYDSEVLY--NKKDGASLGVVSDRLKTLKDVINTSLGRT--DGLEMVHHGADDAN
ExoY-L.Prov.rettg    320 NEQKKLYHNEAEY--NKREGKDNGITNAKIKEINQELNKKLERP--IGLEIVHHGADDAN
CyaA_Bort.pertu.C    249 ARSAVGTEARRQF--RYDGDMNIGVITDFELEVRNALNRRAHAV--GAQDVVQHGTEQNN
EF_Baci.anthr.Cyc    243 IKY--GIERK--------PDSTKGTLSNWQKQMLDRLNEAVKYTGYTGGDVVNHGTEQDN
                                                           EF-switch B/CyaA-loop(H299-K312)

ExoY_Pseu.aerug.1    298 PG-SHMGD--NFPATFYLPRAM--EHRVGEE--------SVRFDEVCVVADRKSFS-LLV
ExoY_Pseu.aerug.2    298 PG-SHMGD--NFPATFYLPRAM--EHRLGEE--------SVRFDEVCVVADRKSFS-LLV
ExoY-L.Prov.stuar    314 PV-TDLDA--NFPATFALPKKI------------------GRFDELCIITNKEELV-ELI
ExoY-L.Burk_pseud    283 PA-TDPGA--NYPATFFLPQKI------------------DRFDEICIIHDERELA-ELI
ExoY-L.Prot.penne    270 PF-AVIHD--NFPATFFVPKSL-LDKPLNSKNQTLDDLFYINNNGTVVLKSPDEFS-KFQ
ExoY-L.Vibr.chole    361 PY-AVMAD--NFPATFFVPKSFFMEDGLGEGKGSIQTYFNVNEQGAVVIRDPHEFS-NFQ
ExoY-L.Vibr.nigri    362 PY-AVMAD--NFPATFFVPKHFFDDDGLGEGKGSIQTYFNVNEQGAVVIQNPQEFS-NFQ
ExoY-L.Vibr.vulni    376 PY-AVMAD--NFPATFFVPKSFFMEDGLGEGKGSIQTYFNVNEQGAVVIRDPQEFS-NFQ
ExoY-L.Prot.vulga    366 PF-AVIHD--NFPATFFVPKSL-LDKPLNSKNQTLDDLFYINNNGTVVLKSPDEFS-KFQ
ExoY-L.Vibr.angui    370 PY-AVMAD--NFPATFFVPKSFFMEDGLGEGKGSIQTYFNVNEQGAVVIRDPQEFS-NFQ
ExoY-L.Prot.mirab    373 PF-SVITD--NFPATFFVPNEL-LDKKLNADNNTLRDFFVVTENNVIIIRDAKEFS-DFQ
ExoY-L.Vibr.ordal    376 PY-AVMAD--NFPATFFVPKSFFMEDGLGEGKGSIQTYFNVNEQGAVVIRDPQEFS-NFQ
ExoY-L.Prov.rettg    376 PG-SVMKD--NFPITFFLPDKLKGKNKLSGTRQSIDTYFQMNLSGAVIINDVESLS-NFQ
CyaA_Bort.pertu.C    305 PF-PEADEKIFVVSATGE-------------------------SQML-TRGQLKEYIG-Q
EF_Baci.anthr.Cyc    293 EEFPEKDNEIFIINPEGE--------------------------FILTKNWEMTGRFIEKN
                         EF-switch B/CyaA-loop(H299-K312)

ExoY_Pseu.aerug.1    344 -ECIKGNGYHFTAHPDWNVPL-RPSFQEALDFFQRKV-----------------------
ExoY_Pseu.aerug.2    344 -ECIKGNGYHFTAHPDWNVPL-RPSFQEALDFSNVRSDARRSLAKPAFPGGMGILPP-VQ
ExoY-L.Prov.stuar    352 -TTSKAEGYHIKTNPLWEKEL-PTIRRPSFEYAKRRLSTVSLNSKITHF-----------
ExoY-L.Burk_pseud    321 -GSAKNAGYHVQINPMWEKEV-ASVQRESFLTARGYFSANTRNIHPLR------------
ExoY-L.Prot.penne    325 -QFMIDLGYIAPINEKWNDGS-NNYFTKKTK-Aâ€”FYHIC-------------------
ExoY-L.Vibr.chole    417 -QVAINVSYRASLNDKWNVGLDDPLFTPKRKL-SHDFLNAKEEVIKKLSGEVETNVRTTQ
ExoY-L.Vibr.nigri    418 -QVAINASYRASLNDKWNSGLDSPLFTTKRKL-SHDYLDARDEVAKKLGLTES--SKL-N
ExoY-L.Vibr.vulni    432 -QVAINVSYRASLNDKWNVGLDDPLFTPKSKL-SHDFLNAKEEVIKKLSGEVETNVRTTQ
ExoY-L.Prot.vulga    421 -QFMIDLGYIAPLNEKWNDGSNNNYFTKKRKL-STTFVDTKKEIGRKFSIDKKDKYTLEY
ExoY-L.Vibr.angui    426 -QVAINVSYRASLNDKWNAGLDDPLFTPKRKL-SHEFLVARDKIAKMLSNESSDLGNDSE
ExoY-L.Prot.mirab    428 -QLVINNGFISPLNEKWNEGIEGNYFSRRRKSSE-NYLNNRELLEKIFSDKNKYKSPLNK
ExoY-L.Vibr.ordal    432 -QVAINVSYRASLNDKWNAGLDDPLFTPKRKL-SHEFLNVRDELANKLKEKAVDFEKEVQ
ExoY-L.Prov.rettg    432 -QLLINEGYRAPLNANWREGEYAEYFNPKRKISDSYINASREIQRKKSIPIAV---RNFI
CyaA_Bort.pertu.C    337 -QRGEGYVFYENRAYGV-AGKSLFDDGLGA------------------------------
EF_Baci.anthr.Cyc    328 -ITGKDYLYYFNRSYNK-IAP-GNKAYIEWTDPITKAKINTIP----------------
```

```
ExoY_Pseu.aerug.1  ------------------
ExoY_Pseu.aerug.2  401 GLPLA-GIC--WAARGS-
ExoY-L.Prov.stuar      ------------------
ExoY-L.Burk_pseud      ------------------
ExoY-L.Prot.penne      ------------------
ExoY-L.Vibr.chole  475 LLTDNE-----GEKLR--
ExoY-L.Vibr.nigri  473 GL---------GERQ---
ExoY-L.Vibr.vulni  490 LLTDNEGLGNEGEKL---
ExoY-L.Prot.vulga  479 ESSLNKFNEGSYIENNSL
ExoY-L.Vibr.angui  484 LEGIAERRTVGSVINHNE
ExoY-L.Prot.mirab  486 INNEYSDYELRLIRSNKL
ExoY-L.Vibr.ordal  490 LDRLGDHQADSTVSTLSV
ExoY-L.Prov.rettg  488 EGKETQSGYIDNIENN--
CyaA_Bort.pertu.C      ------------------
EF_Baci.anthr.Cyc      ------------------
```

Supplementary Figure 8. Sequence alignments of *P. aeruginosa* ExoY, ExoY-like sequences found in Gram-negative bacterial pathogens from the genus *Pseudomonas, Vibrio, Providencia, Burkholderia* or *Proteus* and the related class II nucleotidyl cyclase toxins CyaA from *Bordetella pertussis* and EF from *Bacillus anthracis* (annoted CyaA_Bort.pertu.C, EF_Baci.anthr.Cyc, respectively). NCBI sequence accession numbers and pairwise sequence similarity and identity are indicated in Supplementary Table 2, and Fig.6a displays the phylogenetic tree showing the evolutionary relationships between the aligned sequences. The conserved motifs I and II between ExoY, CyaA and EF that are expected to stabilize the phosphate, ribose and/or base moieties of the bound nucleotide and its divalent cation, the more divergent region III stabilizing the ribose and base moieties of the bound nucleotide and its divalent cation in calmodulin-activated CyaA and EF (EF-switch B/CyaA-loop(H299-K312))[3, 4, 5], and the catalytic deficient mutation K81M in motif I are localized in the sequences.

a

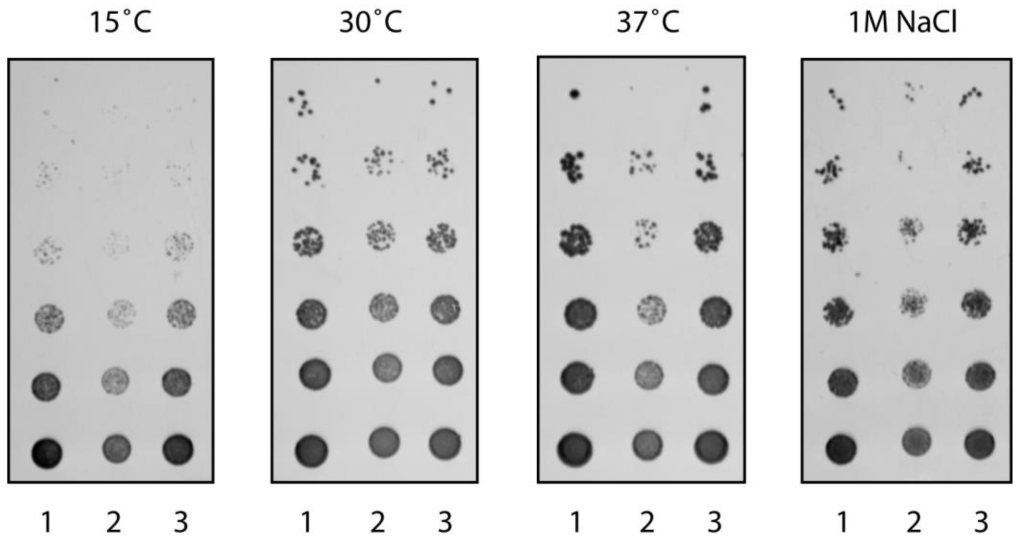

Supplementary Figure 9. Effects of *S. cerevisiae* actin mutant allels on growth under different conditions. SC489 (wt actin, 1), SC690 (D25Y/D222G, 2) and SC691 (D25N, 3) were grown on minimal SD agar plates at 15, 30 or 37ºC or at 30ºC in the presence of 1M NaCl. Cell suspensions were normalized to an OD600 of 1.0 and 5-fold serial dilutions were applied as 3 μl drops on SD agar plates.

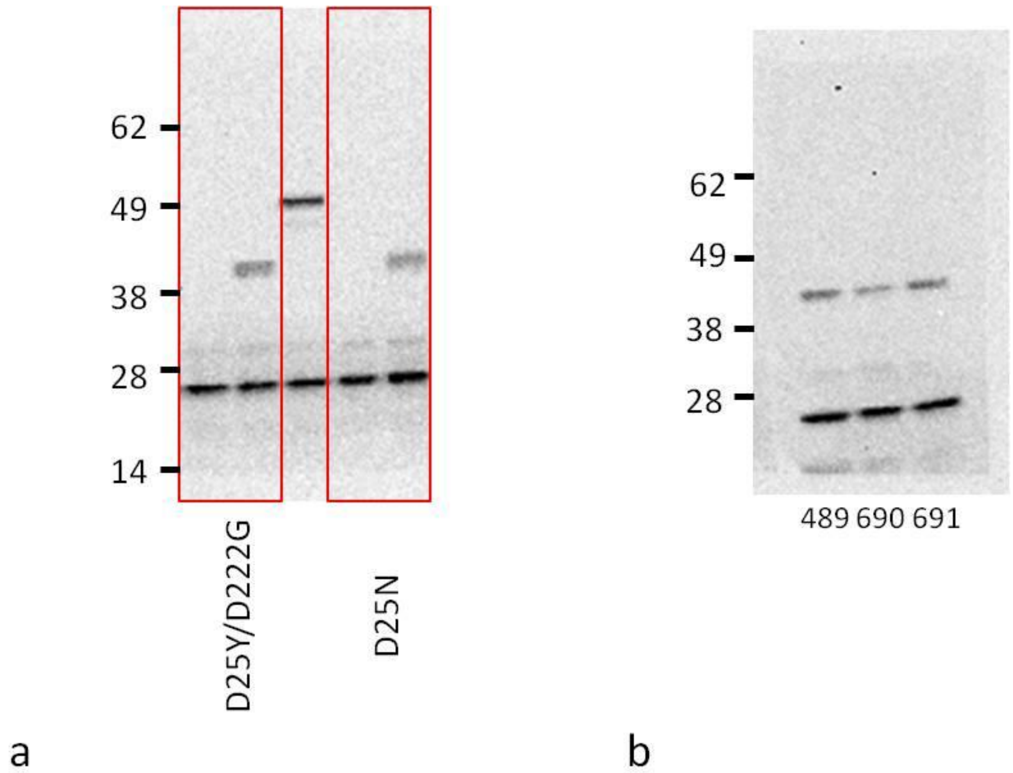

Supplementary Figure 10. Uncropped Western blots of (a) Figure 7b and (b) Figure 7c.

Supplementary Table 1. Strains, plasmids, and primers are described in the table.

	Description	Reference
**strains**		
MG1655	*E. coli K12* wild type	[6]
BLR	*E. coli* BL21, *recA*$^-$: F$^-$*ompThsdS*$_B$(r$_B^-$m$_B^-$) *gal dcm* Δ(srl-recA)306::Tn*10* (Tet^R)	Novagen
BY4741	*Saccharomyces cerevisiae* S288C derivative: Mat a, *his3Δ1 leu2Δ0, met15Δ0, ura3Δ0*	[7]
SC483	haploid *act1::LEU2*(p1177)	[8]
SC489	haploid *act1::LEU2*(p1382)	[8]
SC690	haploid *act1::LEU2*(p1688)	This study
SC691	haploid *act1::LEU2*(p1689)	This study
**plasmids**		
pBAD18	Vector, P$_{ara}$, pBR replicon, Ap^R	[9]
pGEM®-T Easy	Vector for TA cloning	Promega
pDONR221	Gateway vector	Invitrogen
pAcGFP1-N1	vector for expression of proteins fused to the N-terminus of AcGFP in mammalian cells under control of *P*$_{\text{CMV IE}}$	Clontech
pTRCAG	vector expressing CyaC and CyaA from *B. pertussis* under control of a thermoinducible λ promoter	[10]
pAG415GAL-ccdB-TAP	Gateway destination vector: CEN, LEU2, GAL1, TAP	[11]
pAG415GAL-ccdB-HA	Gateway destination vector: CEN, LEU2, GAL1, HA	[11]
pUM447	As pBAD18, HF-ExoY	This study
pUM449	As pBAD18, ExoY-FH	This study
pUM460	inducible expression of ExoY-FH under lambda P$_L$ controlled by temperature sensitive cI (cI857)	This study
pUM478	ExoY entry vector (no stop codon)	This study
pUM482	As pAG415GAL-ccdB-HA, ExoY-HA	This study
pUM483	As pAG415GAL-ccdB-TAP, ExoY-TAP	This study
pUM484	As pUM483, but Hygr^R, ExoY-TAP	This study
pUM485	As pUM482, but Hygr^R, ExoY-HA	This study
pUM497	As pUM484, but ExoY^{K81M}-TAP	This study
pUM498	As pUM485, but ExoY^{K81M}-HA	This study
pUM503	As pUM460, but ExoY^{K81M}-FH	This study
pUM518	As pAcGFP1-N1, ExoY^{K81M}-AcGFP1	This study
pUM522	inducible expression of Vn-ExoY-L-FH under lambda P$_L$ controlled by temperature sensitive cI (cI857)	This study
pEA11	as pGEX-6-P1[12], His-MBP-ExoY-ST	This study
pEA12	as pGEX-6-P1, His-MBP-ExoY^{K81M}-ST	This study
YEpGal555	*E. coli*/*S. cerevisiae* shuttle vector [AmpR/ADE2]	[8]
YCpLac33	*E. coli*/*S. cerevisiae* shuttle vector [AmpR/Ura3]	[13]
p1559	*E. coli*/*S. cerevisiae* shuttle vector [KmR/ADE2]	This study
pRS313	*E. coli*/*S. cerevisiae* shuttle vector [AmpR/HIS3]	[14]
pNL1.1	NanoLuc containing plasmid	Promega
pVK3	derived from pUC4Kan, template for KmR	GE-Healthcare
p1593	Myc-ExoY in YEpGal555	This study
p1595	Myc-ExoY-NanoLuc in YEpGal555	This study
p1654	Myc-ExoY-NanoLuc in p1559	This study

p1177	ACT1 in YCpLac33	[8]
p1182	ACT1 in pRS313	[8]
p1387	ACT1 with deleted ClaI site in pRS313	This study
p1688	$ACT1^{D25Y/D222G}$ in pRS313	This study
p1689	$ACT1^{D25N}$ in pRS313	This study
**primers**		
UM245	5'GGGGAATTCACCATGCGTATCGACGGTCATCG3'	
UM246	5'GGGGCTCGAGGACCTTACGTTGGAAAAAGTCG3'	
UM248	5'GAAAGAATTCACCATGCATCACCATCACCATCACGACTACAAGG ATGACGACGATAAGGGCGGCGGTACCCGTATCGACGGTCATCGTC AGGTGG3'	
UM250	5'GAAACTCGAGCTTGTCATCGTCGTCCTTGTAATCGCCGCCGGTAC CGACCTTACGTTGGAAAAAGTCGAGC3'	
UM254	5'GGGGTCTAGATCAGACCTTACGTTGGAAAAAGTCGAGCG3'	
UM255	5'ACACCATGGGCCGTATCGACGGTCATCGTCAG3'	
UM282	5'AATTAACAAGTTTGTACAAAAAAGCAGGCTTTATGCGTATCGAC GGTCATC3'	
UM283	5'AATTACCACTTTGTACAAGAAAGCTGGGTTGACCTTACGTTGGA AAAAGTCGAGCG3'	
UM302	5'GGAATCCCAACAATTACATCAAAATCCACATTCTCTTCAAAATC AATTGCGGATCCCCGGGTTAATTAA3'	
UM303	5'TCCCGGCATCCGCTTACAGACAAGCTGTGACCGTCTCCGGGA3'	
UM316	5'GGCTCTACAGGACCTGTTC3'	
UM317	5'CTGAGATGCTGGTCGACAC3'	
UM318	5'AGAAGCCCATGGTCGGGAAAC3'	
UM319	5'GTTTCCCGACCATGGGCTTCT3'	
UM345	5'GAGAGCTAGCCACCATGGCGCGTATCGACGGTCATCGTCAG3'	
UM350	5' GGGGAGATCTATGCGTATCGACGGTCATCGT3'	
UM354	5'GCGCCCATGGGCTATAACTATGGTCAGGCTTTGCAGG3'	
UM356	5'CACAGGTACCGAGTCCGTTGAGCTTCGAAGATTC3'	
1259	5' CAGCTCGAGCGTATCGACGGTCATCG 3'	
1260	5' ACAAGGTACCTGGGTTGACCTTACGTTG 3'	
1261	5' TAAAGGTACCATGGTCTTCACACTCG 3'	
1262	5' AGTCGCTAGCTTACGCCAGAATGC 3'	
1217	5' GGAATAAGGGCGCCATGGAAATGTTG 3'	
1218	5' CAACATTTCCATGGCGCCCTTATTCC 3'	
1058	5' CATTTTGAGAATAGATTTGGC 3'	
1059	5' GCCAAATCTATTCTCAAAATG 3'	
1060	5' TTCGTGATAAGTGATAGTG 3'	
M13R	5' CAGGAAACAGCTATGAC 3'	

Supplementary Table 2. Pairwise sequence similarities and identities between ExoY-like sequences found in various Gram-negative bacterial pathogens from the genus *Pseudomonas*, *Vibrio*, *Providencia*, *Burkholderia* or*Proteus* and the related class II nucleotidyl cyclase toxins CyaA from *Bordetella pertussis* and EF from *Bacillus anthracis*.

**Name used in sequence alignement /NCBI Sequence Accession**	**Pathogenic bacterial Genus / individual or RTX toxin (total number of amino-acids)**	**% seq. similarity (identity) with actin-activated *Pseu. aerug.* ExoY**	**% seq. similarity (identity) with actin-activated *Vib. nigrip.* ExoY-like**	**% seq. similarity (identity) with *Bordet. pertussis* CyaA**	**% seq. similarity (identity) with *Bacil. anthracis* EF**
**ExoY_Pseu.aerug.1 / WP_003115517**	***Pseudomonas aeruginosa / individual toxin (378 aa)***	100%	**37.8% (29.4%)**	40.9% (35.8%)	44.3% (37.3%)
ExoY_Pseu.aerug.2 / WP_011666674	*Pseudomonas aeruginosa / individual toxin (414 aa)*	92.5% (91.9%)	36.7% (28.3%)	34.2% (29.4%)	38.9% (31.8%)
ExoY-L.Prov.stuar / WP_040133136	*Providencia stuartii / individual toxin (398 aa)*	53.4% (45.9%)	37.6% (29.6%)	40.5% (33.0%)	42.5% (35.3%
ExoY-L.Burk_pseud / WP_038788970	*Burkholderia pseudomallei / individual toxin (366 aa)*	53.2% (47.1%)	39.2% (29.6%)	45.3% (38.5%)	48.7% (41.8%)
ExoY-L.Prot.penne / EEG85446	*Proteus penneri / individualtoxin (359 aa)*	50.9% (43.0%)	51.8% (44.1%)	45.5% (37.3%)	44.4% (38.7%)
ExoY-L.Vibr.chole / WP_000517829	*Vibrio cholera / MARTX toxin (4405 aa)*	38.9% (29.2%)	79.9% (74.9%)	27.1% (19.0%)	26.3% (20.0%)
**ExoY-L.Vibr.nigri / WP_013610353**	***Vibrio nigripulchritudo* / MARTX toxin (4990 aa)**	**38.0% (29.4%)**	100%	27.8% (19.5%)	28.0% (21.7%)
ExoY-L.Vibr.vulni / WP_039507922	*Vibrio vulnificus / MARTX toxin* (5208 aa)	35.3% (25.6%)	78.0% (71.9%)	24.0% (15.8%)	23.7% (17.4%)
ExoY-L.Prot.vulga / CRL61078	*Proteus vulgaris / RTXtoxin* (2362 aa)	34.4% (26.7%)	53.0% (42.8%)	24.7% (16.5%)	24.7% (18.8%)
ExoY-L.Vibr.angui / WP_019280936	*Vibrio anguillarum / MARTX toxin (4562 aa)*	35.3% (26.2%)	77. 6% (72.6%)	24.4% (16.3%)	23.8% (17.6%)
ExoY-L.Prot.mirab / AGS58688	*Proteus mirabilis / MARTXtoxin* (4083 aa)	34.0% (26.0%)	51.8% (40.9%)	25.1% (16.1%)	24.9% (17.7%)
ExoY-L.Vibr.ordal / WP_010319615	*Vibrio ordalii / RTX toxin* (3916 aa)	34.8% (25.3%)	77.2% (71.3%)	23. 7% (15.2%)	22.9% (16.8%)
ExoY-L.Prov.rettg / EKT57891	*Providencia rettgeri/ RTX toxin* (2370 aa)	33.7% (25.3%)	49.3% (39.6%)	24.6% (15.9%)	24.7% (17. 6%)
CyaA_Bort.pertu.C / J7QLC0	*Bordetellapertussis/ RTXtoxin* (1706 aa)	40.9% (35.8%)	27.8% (19.5%)	100%	53.4% (45.0%)
EF_Baci.anthr.Cyc / P40136	*Bacillus anthracis/ multi-domaintoxin* (800 aa)	44.3% (37.3%)	28.0% (21.7%)	53.4% (45.0%)	100%

Similarity and identity values were computed based on the Supplementary Fig. 8 sequence alignement, and given the BLOSUM62 substitution matrix. The sequence similarities and identities (%) highlighted in green and cyan underline which bacterial nucleotidyl cyclase sequence are the most significantly related to *P. aeruginosa* ExoY or *Vibrio nigripulchritudo* ExoY-like sequence, respectively, and thus may be activated by actin. In gray are highlighted for comparison the similarities between the nucleotidyl cyclase catalytic domain sequences of CyaA from *Bordetella pertussis* and EF from *Bacillus anthracis* that are both activated by Calmodulin. RTX stands for Repeats-in-Toxin, such as glycine and aspartate-rich repeats located at the C-terminus of the toxin proteins, which facilitate export by bacterial secretion systems (T1/3SS), and MARTX for: Multifunctional-Autoprocessing RTX.The phylogenetic tree showing the evolutionary relationships between the aligned sequences is shown in Fig. 6a.

Supplementary References

1. Yeoh S, Pope B, Mannherz HG, Weeds A. Determining the differences in actin binding by human ADF and cofilin. *J Mol Biol* **315**, 911-925 (2002).

2. McGhie EJ, Hayward RD, Koronakis V. Control of actin turnover by a salmonella invasion protein. *Mol Cell* **13**, 497-510 (2004).

3. Drum CL, *et al.* Structural basis for the activation of anthrax adenylyl cyclase exotoxin by calmodulin. *Nature* **415**, 396-402 (2002).

4. Guo Q, Shen Y, Lee YS, Gibbs CS, Mrksich M, Tang WJ. Structural basis for the interaction of Bordetella pertussis adenylyl cyclase toxin with calmodulin. *EMBO J* **24**, 3190-3201 (2005).

5. Yahr TL, Vallis AJ, Hancock MK, Barbieri JT, Frank DW. ExoY, an adenylate cyclase secreted by the Pseudomonas aeruginosa type III system. *Proc Natl Acad Sci U S A* **95**, 13899-13904 (1998).

6. Bachmann B. Derivations and genotypes of some mutant derivatives of Escherichia coli K-12. In: *Escherichia coli and Salmonella: Cellular and Molecular Biology* (ed^(eds). ASM Press (1996).

7. Brachmann CB, *et al.* Designer deletion strains derived from Saccharomyces cerevisiae S288C: a useful set of strains and plasmids for PCR-mediated gene disruption and other applications. *Yeast* **14**, 115-132 (1998).

8. Belyy A, Tabakova I, Lang AE, Jank T, Belyi Y, Aktories K. Roles of Asp179 and Glu270 in ADP-Ribosylation of Actin by Clostridium perfringens Iota Toxin. *PLoS One* **10**, e0145708 (2015).

9. Guzman LM, Belin D, Carson MJ, Beckwith J. Tight regulation, modulation, and high-level expression by vectors containing the arabinose PBAD promoter. *J Bacteriol* **177**, 4121-4130 (1995).

10. Gmira S, Karimova G, Ladant D. Characterization of recombinant Bordetella pertussis adenylate cyclase toxins carrying passenger proteins. *Res Microbiol* **152**, 889-900 (2001).

11. Alberti S, Gitler AD, Lindquist S. A suite of Gateway cloning vectors for high-throughput genetic analysis in Saccharomyces cerevisiae. *Yeast* **24**, 913-919 (2007).

12. Husson C, Renault L, Didry D, Pantaloni D, Carlier MF. Cordon-Bleu uses WH2 domains as multifunctional dynamizers of actin filament assembly. *Mol Cell* **43**, 464-477 (2011).

13. Gietz RD, Sugino A. New yeast-Escherichia coli shuttle vectors constructed with in vitro mutagenized yeast genes lacking six-base pair restriction sites. *Gene* **74**, 527-534 (1988).

14. Sikorski RS, Hieter P. A system of shuttle vectors and yeast host strains designed for efficient manipulation of DNA in Saccharomyces cerevisiae. *Genetics***122**, 19-27 (1989).

15. Gietz RD, Woods RA. Transformation of yeast by lithium acetate/single-stranded carrier DNA/polyethylene glycol method. *Methods Enzymol***350**, 87-96 (2002).
